# Payment schemes and treatment responses after a demand shock in mental health care

**DOI:** 10.1002/hec.4417

**Published:** 2021-09-07

**Authors:** Rudy Douven, Minke Remmerswaal, Tobias Vervliet

**Affiliations:** ^1^ Division Health Care CPB Netherlands Bureau for Economic Policy Analysis The Hague the Netherlands; ^2^ Health Systems and Insurance (HSI) Erasmus School of Health Policy & Management Erasmus University Rotterdam Rotterdam the Netherlands; ^3^ Department of Economics Tilburg University Tilburg the Netherlands; ^4^ Division Labor Market SEO Amsterdam Economics University of Amsterdam Amsterdam the Netherlands

**Keywords:** mental health care, payment system, physician incentives, treatment outcomes

## Abstract

We study whether two groups of mental health care providers—each paid according to a different payment scheme—adjusted the duration of their patients' treatments after they faced an exogenous 20% drop in the number of patients. For the first group of providers, self‐employed providers, we find that they did not increase treatment duration to recoup their income loss. Treatment duration thresholds in the stepwise fee‐for‐service payment function seem to have prevented these providers to treat patients longer. For the second group of providers, large mental health care institutions who were subject to a budget constraint, we find an average increase in treatment duration of 8%. Prior rationing combined with professional uncertainty can explain this increase. We find suggestive evidence for overtreatment of patients as the longer treatments did not result in better patient outcomes, i.e. better General Assessment of Functioning scores.

## 1 INTRODUCTION

Clever payment schemes for providers in health care are considered a promising way to curb health care expenditures while maintaining good quality of care (McClellan, 2011). There is a growing empirical literature which addresses how provider payments models in health care influence provider behavior (Chandra et al., 2011; Chandra & Skinner, 2012; Christianson & Conrad, 2012; Ellis & McGuire, 1986; Johnson, 2014; McGuire, 2000). Designing a payment system is especially challenging for health services which are supply sensitive with heterogenous, unknown or marginal treatment benefits (Skinner, 2011). In this respect, mental health care is a particularly interesting sector to study as uncertainty and variation in treatments are greater than for other health services, and responses to financial incentives are often exacerbated (Frank & McGuire, 2000). Each payment model may provoke different responses of health care providers. In order to find the best payment system, these provider responses and corresponding patient outcomes must be studied.

Our study relates to the literature which studies how providers respond to exogenous demand shocks (Chandra et al., 2011; Clemens & Gottlieb, 2014; Gruber & Owings, 1996; van Dijk et al., 2013). We study provider responses in the context of Dutch mental health care where the government reduced insurance coverage and increased the level of the deductibles in 2012 and 2013. These policies led to a plausible exogenous drop in the number of patients of approximately 20%.^1^ We study and compare the responses for two groups of providers, each paid by a different payment scheme.

The first group are self‐employed providers who are reimbursed per treatment episode. The size of the payment depends on treatment duration in the shape of a staircase: the tariff per treatment episode is flat with large, discontinuous increasing steps at specific treatment duration thresholds. The second group of providers are large mental health institutions with salaried employees that operate under a budget constraint. We study, both theoretically and empirically, to what extent both types of providers changed their treatment behavior in response to this demand shock.

The starting point of our study is an imperfect agency model (McGuire, 2000) that we extend by including specific features of both groups of providers. With this theoretical model we derive several hypotheses about how both groups of providers respond to the demand shock. Next, we test our hypotheses empirically using a large administrative dataset which contains all treatment episodes for all patients in the secondary curative mental health care in the Netherlands. Our sample period covers 4 years before (2008 to 2011) and 2 years after the demand shock (2012 and 2013).

Surprisingly, we find that self‐employed providers did not recoup some of their income loss by prolonging treatment duration after the shock. The treatment duration thresholds in the fee‐for‐service payment function seem to have prevented longer treatments, as providers can only earn more income if they treat patients substantially longer, up to the next tariff threshold.

The results are different for institutional providers. We find that they increased treatment duration by 8%. Furthermore, we find that the increase in treatment duration did not translate into better treatment outcomes, which suggests overtreatment of the patients. Institutional providers implicitly or explicitly perceived some form of rationing before the demand shock, because when they gained more time per patient due to the demand shock, they increased treatment duration. We argue that the main driver for this increase is not an income effect but provider uncertainty: differences in beliefs, decision‐making, and motivation of providers are important drivers of supply‐side variation (Abaluck et al., 2016; Currie and McLeod, 2017, 2018; Currie et al., 2016; Cutler et al., 2019). Providers expected that the extra treatment time would benefit patients, but these expectations did on average not result in better treatment outcomes. In theory, providing more services will result in better outcomes as long as treatments are not already at flat of the curve (Fuchs, 2004). In this study, we are able to compare each patient's health status before and after treatment to measure an effect of a change in treatment duration. Such comparisons are often difficult in empirical work due to a lack of data (quality), patient heterogeneity, and endogenous provider choices.^2^

Our work has several implications for policy. We confirm earlier findings in the literature that treatments in mental health care are indeed supply sensitive and that patients may be treated longer than necessary (Wennberg, 2010). Implementing financial restrictions to limit treatment duration, as well as implementing objective rules when to stop providing mental health care services (Koekkoek et al., 2019), can be welfare enhancing when carried out in a smart way.

This study complements previous work on the supply‐side of the Dutch mental health care sector. Douven et al. (2015) show that self‐employed providers who were paid according to the stepwise fee‐for‐service payment scheme showed different treatment behavior than budgeted providers between 2008 and 2010. Moreover, altruistic providers treated mental health patient shorter and reported better patient outcomes than financially motivated providers (Douven et al., 2019). In this study, we gathered 3 years of additional data which allows us to study treatment responses of providers after a large demand shock in 2012.

## 2 INSTITUTIONAL SETTING

This study focuses on the secondary curative mental health care sector in the Netherlands. Curative mental health care is specialized care for patients with severe mental health conditions.^3^ It concerns mainly outpatient care, for example, a patient who visits a psychologist or psychiatrist for one or more hours per week for several consecutive weeks or months.

Secondary curative mental health care is part of the basic benefit package and therefore covered by the mandatory insurance scheme for all inhabitants of the Netherlands. Patients need a referral from their general practitioner and face out‐of‐pocket payments for mental health care services: a mandatory generic deductible which applies to most of the services in the basic insurance package. In 2008, this deductible was 150 euros, and it was gradually increased by the government to 350 euros in 2013.

In this study we distinguish two groups of providers according to their reimbursement system: self‐employed providers and budgeted providers. Henceforth, we will name the two groups of providers based on their payment scheme: we refer to self‐employed providers as “FFS‐providers” and budgeted providers as “BUD‐providers.”

Roughly 10% of all treatments are provided by FFS‐providers. FFS‐providers are specialists who work independently or in small groups in small private practices with relatively low investment costs and flexible labor contracts. FFS‐providers are compensated by health insurers for each treatment episode, which is defined by a Diagnosis Treatment Combination (DBC^4^). Every DBC is a treatment episode with a specific diagnosis and a specific treatment.^5^, ^6^ FFS‐providers negotiate a tariff for each DBC with insurers, designed to cover average estimated labor and capital costs for a treatment.^7^ The maximum tariff for each DBC is determined by the Dutch Healthcare Authority (NZa). The tariff for a DBC follows a stepwise fee‐for‐service function with thresholds at 250, 800, 1800, 3000 and 6000 min. Between these thresholds the tariffs are flat. Figure S1 (see Appendix A in Supporting Information S1) shows an example of the tariff structure for providers who treat patients with schizophrenia.

The majority of the treatments, about 90%, are provided by BUD‐providers. BUD‐providers are large mental health institutions such as regional facilities for ambulatory care or specialized psychiatric hospitals. Employees have long‐term contracts, fixed working times and receive a salary. Since BUD‐providers are more specialized, they also attract patients with more severe conditions than FFS‐providers. Importantly, until 2014, these institutions were not compensated according to their DBC production, but based on annual budgets. Budgeted providers did however record all their treatment episodes as DBCs. The differences of the payment systems and financial incentives for FFS‐providers and BUD‐providers will be discussed in more detail in the next section.

Important for our analyses is that there were waiting lists for mental health care in the Netherlands during our sample period. For example, in 2010 adults had to wait on average about 5 weeks to obtain their first intake and an additional 5 weeks to obtain treatment (Dutch Healthcare Authority, 2014). This suggests that many providers may have faced some form of rationing in the pre‐period.

### 2.1 Market developments and the demand shock in 2012 and 2013

To reduce spending, the Dutch government implemented three different types of reforms in 2012 and 2013. Insurance coverage for mental health services was reduced, cost‐sharing for mental health use was increased, and the regulated maximum tariffs for treatment episodes were lowered.

First, the government excluded all treatments from the basic insurance package with a diagnosis “adjustment disorder” in 2012 and “V‐code disorders” in 2013. These two diagnoses covered about 17% of all treatments.^8^ These treatments were no longer covered by the basic benefit package and patients therefore had to pay for the entire treatment out of their own pocket. An important note for our analyses is that—after the policy changes—there are almost no DBCs registered with these diagnoses anymore. This will be discussed further in Section 4.

Second, in 2012, the government introduced a deductible of 200 euros for secondary mental health care specifically. This was on top of a mandatory deductible for all curative care services. One year later, in 2013, the government again abolished the deductible for mental health care, but simultaneously increased the mandatory general deductible from 220 euros in 2012 to 350 euros in 2013.^9^ We assume that the 200 euros deductible for secondary mental health care only affects the extensive margin, and not the intensive margin as the deductible is already exhausted after the first visit and any follow‐up visits are without costs for the patient. This assumption will allow us to relate changes in treatment duration to financial incentives on the supply‐side and not to the demand‐side.

Third, the government lowered maximum tariffs for all treatment episodes with 5.5% in 2012 and 2013 (Dutch Healthcare Authority, 2014). Correspondingly, the total budget for BUD‐providers was lowered with 5.5%. Furthermore, in 2013 the government, insurers and mental health care sector collectively agreed to limit the future growth of curative mental health care spending.

## 3 THEORETICAL FRAMEWORK

To model how providers respond to a shock in demand we build upon the literature on physician agency models (see, e.g., Cutler et al., 2019; Douven et al., 2019; McGuire and Pauly, 1991). We extend these models in several ways. First, we allow for supply side‐variation and we model professional uncertainty that is a result of differences among providers in beliefs, decision‐making, and motivation (Chandra & Skinner, 2012). Second, we allow that providers and patients may differ in how they perceive the value of a treatment. Third, for both types of providers we explicitly model the key characteristics of their payment function.

### 3.1 General model

In our model, a provider j decides on treating a patient i. The key instrument in this decision is the duration of the treatment episode $x_{i}$, measured in minutes.

The demand‐side of the model is an indirect patient utility function which is a function of a patient's health status, his or her out‐of‐pocket payments, preferences, and the duration of his or her treatment. It reflects the demand of a fully informed patient (Cutler et al., 2019). In this function, we denote the patient's “true” benefit from a treatment or the quality of a treatment as $Q_{i}$ with $Q_{i}^{\prime \prime }\left(x_{i}\right)\leq 0$.^10^ Solving the demand function for optimal treatment duration yields $x_{i}^{D}$, which we assume is the fully informed patient's demand. Overtreatment occurs if $x_{i}>x_{i}^{D}$ and $Q_{i}\left(x_{i}\right)-Q_{i}\left(x_{i}^{D}\right)\leq 0$, that is, for additional care provided at the margin, a patient gains no improvements in health.

At the supply‐side, a provider's utility from treating patients, each patient with a mental health status $\theta_{i}$, depends on two components: the utility a provider j attaches to perceived patient benefits, denoted by $S_{ij}$, and the utility from net financial benefits, denoted by $\Pi_{j}$:

(1) $$U_{j}\left(x,\theta \right)=\sum_{i=1}^{q}\;S_{ij}\left(x_{i},\theta_{i}\right)+\alpha_{j}\Pi_{j}$$

We allow $S_{ij}$, with $S_{ij}^{\prime \prime }\left(x_{i}\right)\leq 0$, to differ across providers because of professional uncertainty. $S_{ij}\left(x_{i}\right)-S_{ij}\left(x_{i}^{D}\right)>0$ and $Q_{i}\left(x_{i}\right)-Q_{i}\left(x_{i}^{D}\right)\leq 0$ may both hold: some providers may believe it is beneficial to treat patients longer but in fact overtreat from a patient's perspective. Net financial benefits $\Pi_{j}$ is:

(2) $$\Pi_{j}\left(x,\theta \right)=\sum_{i=1}^{q}\left(p_{i}\left(x_{i},\theta_{i}\right)-{vc}_{j}x_{i}\right)-fc_{j}$$

where $p_{i}\left(x_{i},\theta_{i}\right)$ is the payment per individual treatment episode, which we will define more precisely below. Total costs are the sum of two components: variable costs ${vc}_{j}$ proportional to $x_{i}$, and fixed costs ${fc}_{j}$. A provider maximizes utility (1):

(3) $$\max_{x_{i}}\;\sum_{i=1}^{q}\;S_{ij}\left(x_{i},\theta_{i}\right)+\alpha_{j}\Pi_{j}\left(x,\theta \right)$$

$$such\;that\;\Pi_{j}\left(x,\theta \right)\geq 0$$

The main difference between both types of providers is their payments system. We use superscripts FFS and BUD to distinguish between both types.

### 3.2 Fee‐for‐service providers

FFS‐providers receive a financial compensation per treatment episode i which resembles a stepwise (or staircase) fee‐for‐service function:

(4) $$p_{i}^{\text{FFS}}\left(x_{i},\theta_{i}\right)=P_{i}\left(k^{l},\theta_{i}\right)\;for\;k^{l}\leq x_{i}<k^{l+1}$$

where $k^{l}$ represents a treatment duration threshold at l minutes. To obtain the optimal treatment duration $x_{i}^{*,FFS}$ we use a Lagrange multiplier λ:

(5) $$\frac{\partial S_{ij}^{\text{FFS}}\left(x_{i}^{*,FFS},\theta_{i}\right)}{\partial x_{i}}=\left(\alpha_{j}^{\text{FFS}}-\lambda \right)vc_{j}^{\text{FFS}}\;or\;x_{i}^{*,FFS}=k^{l}$$

$$and\;\Pi_{j}^{*,FFS}\left(x^{*,FFS},\theta \right)\geq 0$$

After the reform the number of patients dropped with about 20%, that is, q drops to about 0.8q. Prices were cut by 5.5%, that is, $p_{i}^{\text{FFS}}$ drops to $0.945p_{i}^{\text{FFS}}$. In general, a fall in price has an income effect and a substitution effect (McGuire & Pauly, 1991), but in our case the substitution effect disappears because $\partial p_{i}^{\text{FFS}}/\partial x_{i}=0$ due to the stepwise payment scheme. After the shock, the new optimum $x_{i}^{**,FFS}$ satisfies:

(6) $$\frac{\partial S_{ij}^{\text{FFS}}\left(x_{i}^{**,FFS},\theta_{i}\right)}{\partial x_{i}}=\left(\alpha_{j}^{\text{FFS}}-\lambda \right)vc_{j}^{\text{FFS}}\;or\;x_{i}^{**,FFS}=k^{l}$$

$$and\;\Pi_{j}^{**,FFS}\left(x^{**,FFS},\theta \right)=\sum_{i=1}^{0.8q}\left(0.945p_{i}^{\text{FFS}}\left(x_{i}^{**,FFS},\theta_{i}\right)-vc_{j}^{\text{FFS}}x_{i}^{**,FFS}\right)-fc_{j}^{\text{FFS}}\geq 0$$

Comparing Equation (6) with Equation (5), we observe that only the inequality restriction changes. Thus FFS‐providers will only increase treatment duration after the reform if their income, due to fewer patients and lower prices, does not cover total variable and fixed costs. For example, if fixed costs are low FFS‐providers will treat their patients in the same way as before.

The model above does not capture that some providers may treat their patients longer to recoup some of their lost income after the shock, even if total costs are covered. This idea is similar to the target or reference income hypothesis (McGuire & Pauly, 1991; Rizzo & Zeckhauser, 2003). We model this by including $\gamma_{j}$ in Equation (1):

(7) $$U_{j}=\sum_{i=1}^{q}\;S_{ij}\left(x_{i},\theta_{i}\right)+\alpha_{j}\Pi_{j}^{\frac{1}{\gamma_{j}}}$$

When $\gamma_{j}=1$ the model is the same as before, but if $\gamma_{j}>1$ the weight on net financial benefits increases when these benefits are low. Thus, if FFS‐providers lose some of their income then their incentives increase to recoup some of this lost income. Under mild regularity conditions, the optimal solution is given by:

(8) $$\frac{\partial S_{ij}^{\text{FFS}}\left(x_{i}^{*},\theta_{i},q\right)}{\partial x_{i}}={\bar{\alpha_{j}}}^{\text{FFS}}vc_{j}^{\text{FFS}}\;or\;x_{i}^{*,FFS}=k^{l}$$

with

$${\bar{\alpha_{j}}}^{\text{FFS}}=\frac{\alpha_{j}^{\text{FFS}}}{\gamma_{j}\;}{\left(\Pi_{j}^{*,FFS}\right)}^{\frac{1-\gamma_{j}}{\gamma_{j}}}$$

The only difference with Equation (5) is the term ${\bar{\alpha_{j}}}^{\text{FFS}}$. The larger $\gamma_{j}$ is, the smaller ${\bar{\alpha_{j}}}^{\text{FFS}}$, and the more FFS‐providers will increase their treatment durations and get closer to the “flat of the curve”, as marginal patient benefit $\partial S_{ij}^{\text{FFS}}/\partial x_{i}$ declines. This holds only for financially motivated providers who care (strongly) about their income ($\alpha_{j}^{\text{FFS}}\gg 0$) and not for altruistically motivated providers ($\alpha_{j}^{\text{FFS}}=0$).

Hypothesis 1 **FFS‐providers** FFS‐providers have an incentive to increase treatment duration after the shock if their income becomes negative. Financially motivated FFS‐providers ($\alpha_{j}^{\text{FFS}}\gg 0$ and $\gamma_{j}>1$) will increase treatment duration to recoup some of their lost income.

Note that for FFS‐providers undertreatment is not an issue as they are unconstrained in how long they can treat patients. This may be different for BUD‐providers. Due to budgets there may have been implicit or explicit rationing before the shock. After the demand shock, the relative capacity per patient increases which allows them to reveal, or to get closer to, their unconstrained optimum.

### 3.3 BUD‐providers

BUD‐providers operate under a budget constraint $Y_{j}^{\text{BUD}}$ that is determined ex‐ante after a negotiation process with the representative health insurer in the region. In practice, these budget negotiations are private and unobserved. Several factors play a role in these negotiations such as the previous year's budget, the patients' mental conditions along with other undetermined factors. At the employee level, psychologists and psychiatrists are salaried and have fixed working times which may constrain their available time per patient. Also there is no directly observable price tag for an individual treatment episode i. Therefore, we approximate it by price $p_{i}^{\text{BUD}}\left(x_{i},\theta_{i}\right)$, with $\partial p_{i}^{\text{BUD}}/\partial x_{i}\geq 0$. A budget implies that a BUD‐provider j can produce q treatment episodes up to a ceiling $Y_{j}^{\text{BUD}}$:

(9) $$\sum_{i=1}^{q}\;p_{i}^{\text{BUD}}\left(x_{i},\theta_{i}\right)\leq Y_{j}^{\text{BUD}}$$

If the above inequality is strict providers receive less than the budget $Y_{j}^{\text{BUD}}$. Providers who exceed the budget receive $Y_{j}^{\text{BUD}}$, and will not be reimbursed for the additional production. Here we assume a hard budget constraint, but in our discussion section we will interpret our results also in the light of a soft budget constraint. BUD‐providers maximize their utility:

(10) $$\max_{x_{i}}\;\sum_{i=1}^{q}\;S_{ij}^{\text{BUD}}\left(x_{i},\theta_{i}\right)+\alpha_{j}^{\text{BUD}}\left\{\sum_{i=1}^{q}\left(\left.p_{i}^{\text{BUD}}\left(x_{i},\theta_{i}\right)-vc_{j}^{\text{BUD}}x_{i}\right)-fc_{j}^{\text{BUD}}\right.\right\}$$

$$subject\;to\;fc_{j}^{\text{BUD}}+\sum_{i=1}^{q}\;vc_{j}^{\text{BUD}}x_{i}\leq \sum_{i=1}^{q}\;p_{i}^{\text{BUD}}\left(x_{i},\theta_{i}\right)\leq Y_{j}^{\text{BUD}}$$

The difference with FFS‐providers is the different payment $p_{i}^{\text{BUD}}$ and the two‐sided inequality constraint due to the budget. Using Lagrange multipliers $\lambda_{1}$ and $\lambda_{2}$ yields:

(11) $$\frac{\partial S_{ij}^{\text{BUD}}\left(x_{i}^{*,BUD},\theta_{i}\right)}{\partial x_{i}}+\left(\alpha_{j}^{\text{BUD}}-\left(\lambda_{1}+\lambda_{2}\right)\right)\frac{\partial p_{i}^{\text{BUD}}\left(x_{i}^{*,BUD},\theta_{i}\right)}{\partial x_{i}}=\left(\alpha_{j}^{\text{BUD}}-\lambda_{1}\right)vc_{j}^{\text{BUD}}\;$$

$$and\;fc_{j}^{\text{BUD}}+\sum_{i=1}^{q}vc_{j}^{\text{BUD}}x_{i}^{*,BUD}\leq \sum_{i=1}^{q}p_{i}^{\text{BUD}}\left(x_{i}^{*,BUD},\theta_{i}\right)\leq Y_{j}^{\text{BUD}}$$

There are several solutions. For example, $\lambda_{1}=\lambda_{2}=0$ reflects the global optimum as both restrictions are not binding. $\lambda_{1}>0$, $\lambda_{2}=0$ reflects the case were providers treat patients longer than in the global optimum to obtain zero net financial benefits. $\lambda_{1}=0$, $\lambda_{2}>0$ reflects rationing; providers do not treat patients longer because they are constrained by the budget.

After the reform q drops to about 0.8q and $p_{i}^{\text{BUD}}$ to $0.945p_{i}^{\text{BUD}}$. We assume the budget was lowered with about 5.5% (see Section 2), thus $Y_{j}^{\text{BUD}}$ drops to $0.945Y_{j}^{\text{BUD}}$. After the shock, the optimum $x_{i}^{**,BUD}$ satisfies:

(12) $$\frac{\partial S_{ij}^{\text{BUD}}\left(x_{i}^{**,BUD},\theta_{i}\right)}{\partial x_{i}}+0.945\left(\alpha_{j}^{\text{BUD}}-\left(\lambda_{1}+\lambda_{2}\right)\right)\frac{\partial p_{i}^{\text{BUD}}\left(x_{i}^{**,BUD},\theta_{i}\right)}{\partial x_{i}}=\left(\alpha_{j}^{B}-\lambda_{1}\right)vc_{j}^{\text{BUD}}\;$$

$$and\;fc_{j}^{\text{BUD}}+\sum_{i=1}^{0.8q}\;vc_{j}^{\text{BUD}}\leq 0.945\sum_{i=1}^{0.8q}\;p_{i}^{\text{BUD}}\left(x_{i}^{**,B},\theta_{i}\right)\leq 0.945Y_{j}^{\text{BUD}}$$

Comparing both solutions yields that there now is a substitution effect as $\partial p_{i}^{\text{BUD}}/\partial x_{i}\geq 0$. Due to a drop in price providers have an incentive to reduce treatment ($x_{i}^{*,BUD}>x_{i}^{**,BUD}$) because leisure becomes more valuable. The other main aspect in our model is rationing. In a situation of prior rationing ($\lambda_{1}=0,\lambda_{2}>0$), providers receive additional room (in terms of finance and time) to treat patients longer after the shock ($x_{i}^{*,BUD}<x_{i}^{**,BUD}$). Longer treatments may enhance provider utility because BUD‐providers may find it beneficial for the patient and/or it may increase their income at the institutional level.

Hypothesis 2 **BUD‐providers** After the shock, BUD‐providers have an incentive to reduce treatment duration if the substitution effects dominates. BUD‐providers have an incentive to increase treatment duration if there was either prior rationing and providers believe it is beneficial for their patients or/and providers want to increase their income (budget).

### 3.4 Testing for overtreatment

Treating similar patients longer or shorter after the shock could be both welfare enhancing or decreasing. Note that in our theoretical model we use for patient benefit the provider perspective S while for measuring welfare we need the patient perspective Q. Since we assume ${Q_{i}}^{\prime \prime }\left(x_{i}\right)\leq 0$ we can only credibly test for overtreatment.

Hypothesis 3 **overtreatment** Let d= FFS,BUD. We have suggestive evidence for overtreatment of mental health services if providers prolong treatment duration after the demand shock, that is, $x_{i}^{**,d}>x_{i}^{*,d}$, and $Q\left(x_{i}^{**,d}\right)-Q\left(x_{i}^{*,d}\right)\leq 0$ or if providers decrease treatment duration after the shock, that is, $x_{i}^{**,d}<x_{i}^{*,d}$ and $Q\left(x_{i}^{**,d}\right)-Q\left(x_{i}^{*,d}\right)\geq 0$

## 4 DESCRIPTION OF THE DATA

We use a proprietary administrative dataset obtained from the Dutch healthcare regulator, the Dutch Healthcare Authority (NZa), that contains detailed information on all treatments in the curative Dutch mental health care sector. The data include patient characteristics, provider characteristics, treatment characteristics, and treatment outcomes.

For each patient, the diagnosis, consisting of a main and sub‐diagnosis, is registered by the provider. To illustrate, we can observe that a patient has a “mood” disorder with sub‐diagnosis “Depression,” and for example not a “bipolar” disorder. There are 19 main diagnoses and over a hundred sub‐diagnoses. At the beginning of a treatment episode, each practitioner assesses the mental health status of a patient by means of the Global Assessment of Functioning (GAF). This start GAF score is measured on a 10‐point scale, where lower GAF scores indicate more severe mental health conditions and higher GAF scores imply less severe conditions.^11^ For each patient also age and gender is available.

Providers are grouped into FFS‐ and BUD‐providers (see also Section 2). Using a unique provider ID, we can follow all treatment episodes per provider over the 6‐year period.

For each treatment episode several treatment characteristics are recorded. The exact day of the start and end of a treatment episode are recorded. It is possible that a treatment episode is finished, but the treatment is not, for example when the treatment lasts longer than 365 days. In that case, a provider starts a new treatment episode for administrative reasons labeled “continued treatment.” The prior treatment episode is then labeled “regular treatment.” Regular and continued treatments comprise over 90% of all treatments episodes. Providers record each minute they spend on a patient in the treatment episode. As a result, we observe treatment duration per treatment episode, which is one of the main dependent variables in our empirical analysis. Furthermore, providers distinguish between direct treatment time, when a provider is treating the patient in the actual presence of the patient, and indirect treatment time, when the provider is doing preparation or administrative work for the patient.

The data also offer treatment outcomes: the improvement in mental health status during the treatment episode. This is the difference between the start GAF score and the GAF score at the end of a treatment episode, which we will henceforth refer to as *DIFGAF*, another key outcome variable in the empirical analysis.

We performed several data cleaning and sample selection steps which we explain in Appendix B (see Supporting Information S1). The final sample consists of 740 FFS‐providers with in total 253,261 treatment episodes and 357 BUD‐providers with 3,893,294 treatment episodes. Thus, BUD‐providers account for a large majority of all treatment episodes. The average number of treatment episodes per BUD‐provider is roughly 11,000 compared to 342 for FFS‐provider (about a 30 times difference). Because both provider groups differ in size, type of payment system and type of patients, we analyze them separately.

### 4.1 Descriptive statistics

**FIGURE 1:**
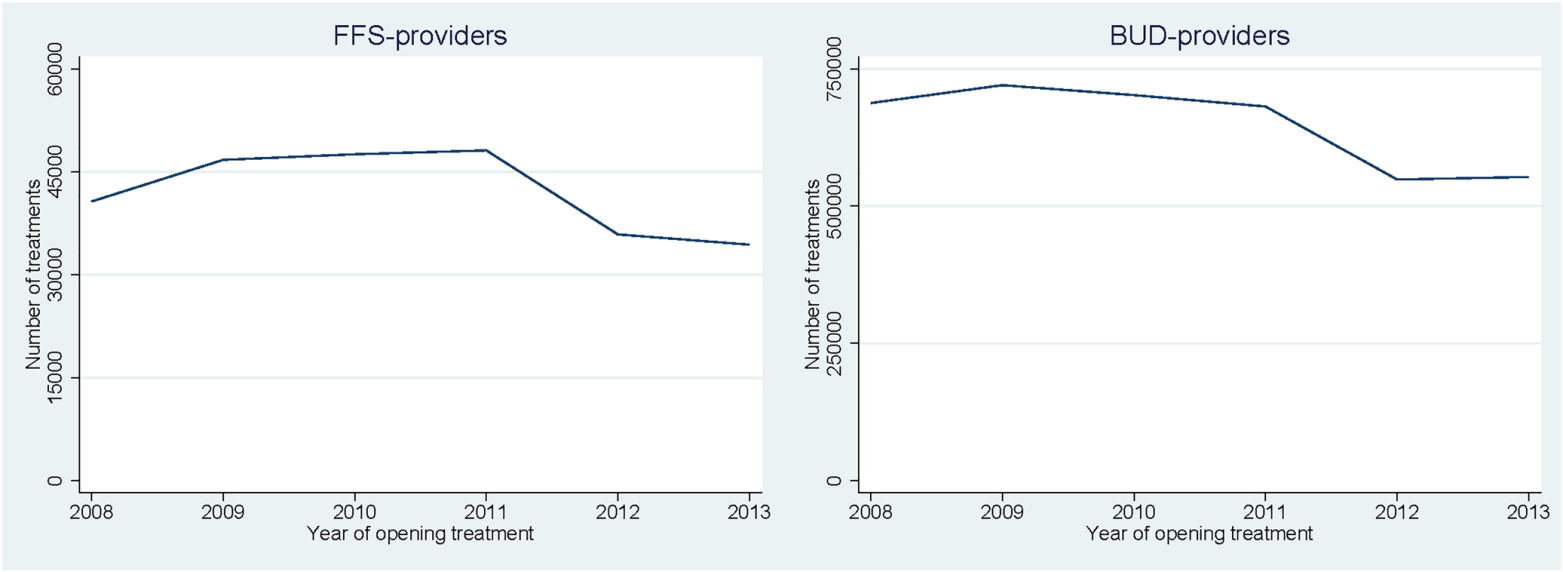
The total number of treatment episodes per year

The two panels in Figure 1 show the number of treatment episodes for FFS‐ and BUD‐providers between 2008 and 2013. Up to 2011 the number of treatment episodes did not change much for both provider types. Then, in 2012, we clearly observe the demand shock. For FFS‐providers, the number of treatment episodes drop in 2012 with roughly 22% compared to 2011 and for BUD‐providers the drop is about 20%. The lower number of treatment episodes of 2012 persists in 2013.

Tables S2 and S3 (see Appendix C in Supporting Information S1) present information for the five largest diagnosis groups. We find mixed results. For some diagnoses, there is a drop in the number of treatment episodes in 2012 and 2013 while for others there is no decrease or even a slight increase. Diagnosis groups “adjustment” disorders and “V‐codes” exhibit remarkable patterns over time: the drop is so large that there are almost no treatment episodes left in 2013. This is the result of the removal of both diagnoses from the basic benefit package, as we explained in Section 2. For prevalent diagnoses, such as “mood,” “personality” and “anxiety” disorders, we observe hardly any drop in the number of treatment episodes in 2012 and 2013.

**FIGURE 2:**
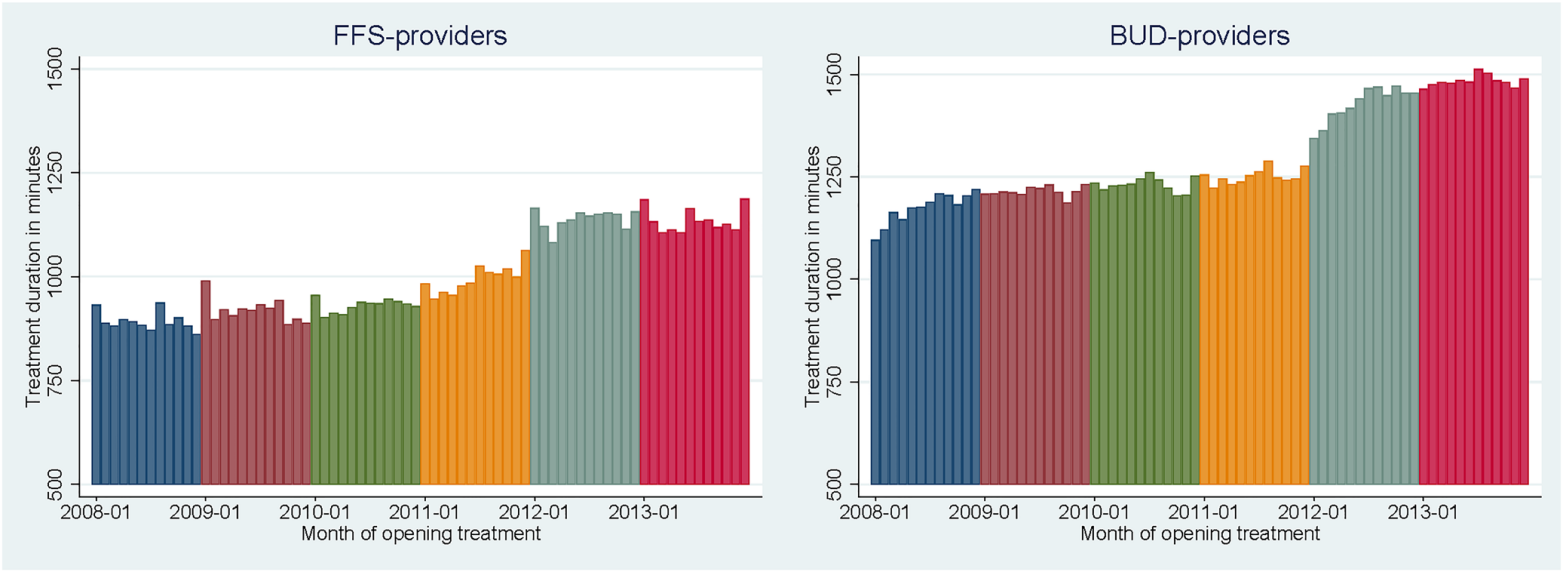
Average treatment duration per treatment episode per month (months are plotted on the horizontal axis with 2008‐01 as January 2008)

For both types of providers, average treatment duration per treatment episode increased in 2012 and 2013. This increase is clearly visible in Figure 2. For FFS‐providers, treatment duration increased on average from 995 min in 2011 to 1138 min in 2012 and 2013; an increase of about 14%. For BUD‐providers, the increase is about 16%, from 1251 to 1455 min on average. Note that the absolute increase in treatment duration is considerably larger for BUD‐providers than for FFS‐providers. Average treatment duration in Tables S2 and S3 (see Appendix C in Supporting Information S1) show that the increase is distributed homogeneously over all start GAF categories.

**FIGURE 3:**
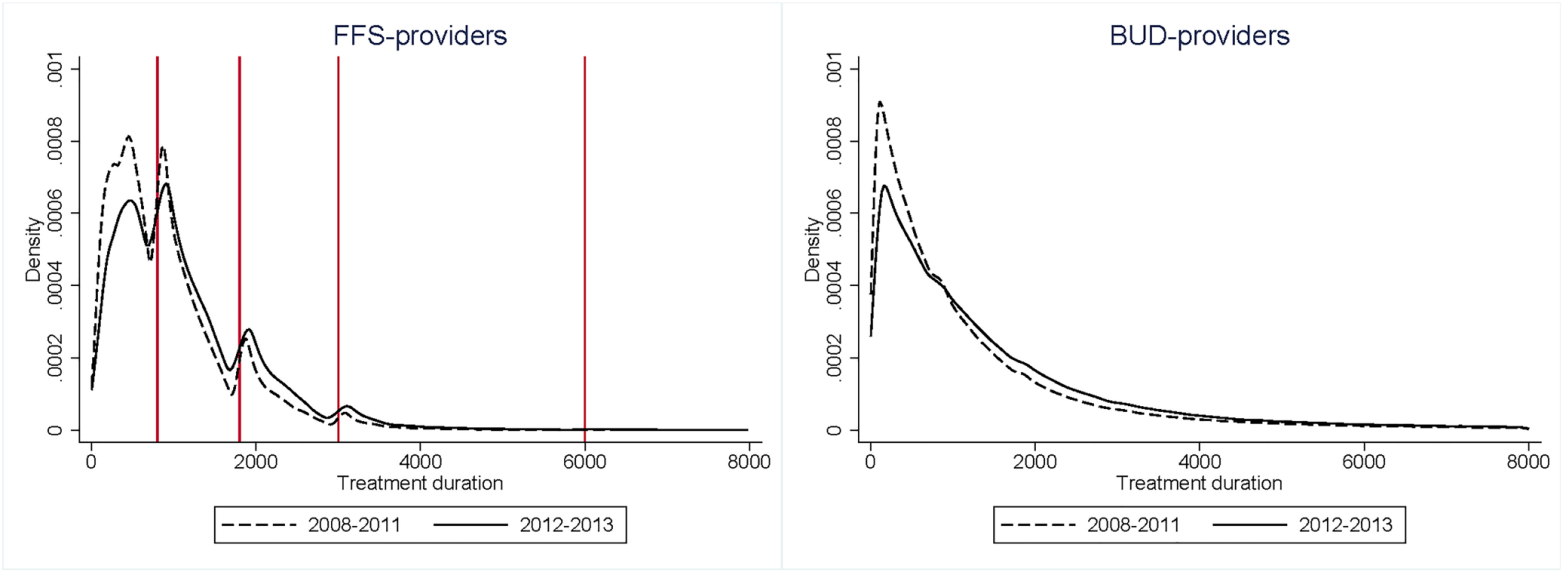
Distribution of treatment duration before and after the demand shock

The distribution of treatment duration between FFS‐ and BUD‐providers differs greatly, which is the result of their different payment models. Figure 3 shows the distribution of both types of providers before and after the demand shock. There are gaps before and bunches just after treatment duration thresholds (indicated in Figure 3 by the vertical lines) in the distribution of FFS‐providers. FFS‐providers exploit the thresholds in the stepwise fee‐for‐service function to end a treatment just after a treatment duration threshold, as this increases their reimbursement considerably (see Section 3). In our empirical strategy, we will follow Douven et al. (2019) and exploit this variation around the thresholds to separate financially from altruistically motivated FFS‐providers. Financially motivated FFS‐providers will exploit the thresholds while altruistically motivated providers do not react to these thresholds. In contrast, the treatment duration distribution of BUD‐providers is relatively smooth with a mass between 250 and 2000 min. The distribution is smooth because BUD‐providers' income is mainly determined by a budget and the fact that employees receive a salary. Their payment does not depend as much on the duration of a single treatment. Figure 3 shows that the distribution for both FFS‐ and BUD‐providers is more skewed to the right in the post‐period, which reflects an increase in average treatment duration in 2012–2013.

**FIGURE 4:**
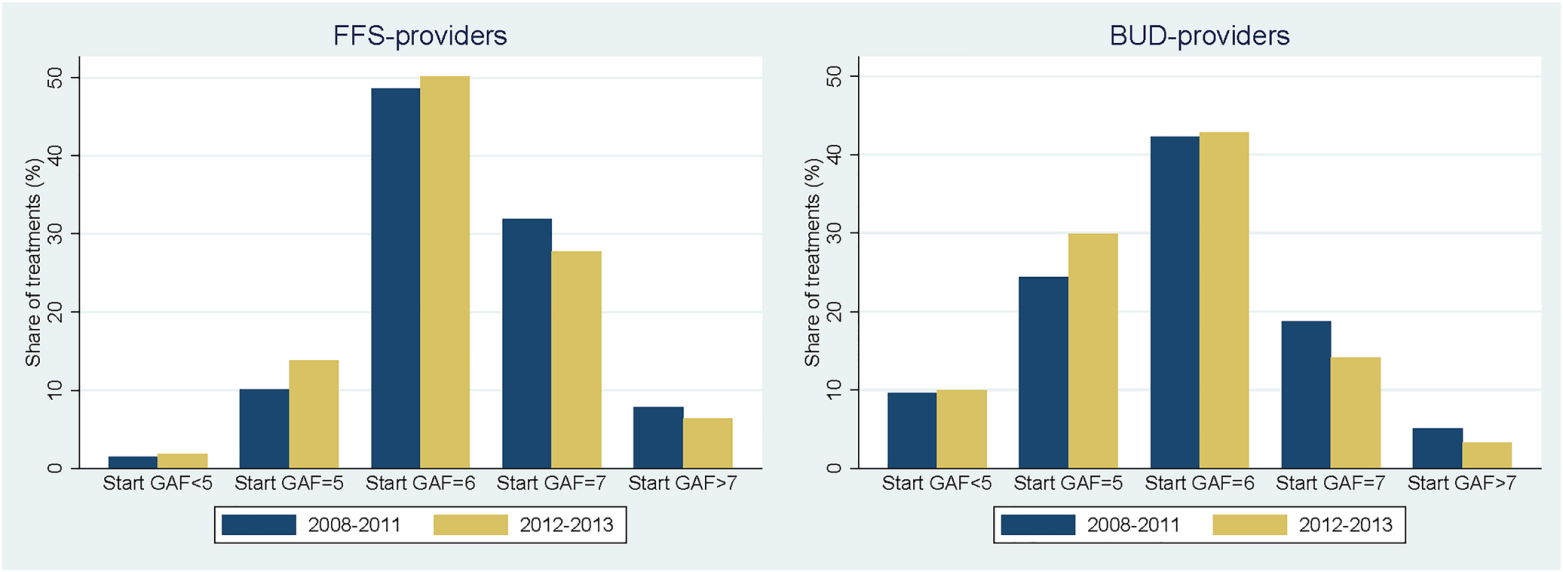
Relative change in start Global Assessment of Functioning (GAF) scores

Persons with a more severe mental condition (i.e., lower GAF‐score) are on average treated longer. For example, patients with a start GAF<5 are treated on average about three times longer than patients with a start GAF>7. FFS‐providers have on average less severe patients than BUD‐providers. Figure 4 shows how the start GAF‐scores change before and after the demand shock. For both, FFS‐ and BUD‐providers, the distribution of start GAF‐scores are more skewed to the left in the post‐period, which indicates that both types of providers treat relatively more severe patients after the shock in 2012–2013. An important reason is that the government excluded “adjustment disorders” and “V‐codes” from the basic benefit package which are relatively mild health conditions compared to depression, anxiety disorder etc. Another reason might be that relatively less severe patients forego more treatments after the introduction of the deductible for mental health care in 2012. Since the deductible is independent of the severity of a patient's illness, patients with a mild health condition might find a treatment less beneficial than patients with a more severe health condition. Because patients with a mild health condition have relatively shorter treatments it is important to control for case‐mix as otherwise this effect would be picked up as an exacerbated effect of the demand shock (see also Lambregts & van Vliet, 2018; Ravesteijn et al., 2017).

## 5 ESTIMATION METHODOLOGY

The goal of our empirical approach is to analyze whether providers changed treatment duration for similar patients in 2012 and 2013 compared to the years before. We will first explain the general idea of our methodology which we apply to multiple dependent variables to estimate various responses to the demand shock such as the drop in the number of treatment episodes, the effect on treatment duration, and the effect on patient outcomes (respectively, q, $x_{i}$ and $Q_{i}$ in the theoretical framework).

Since we lack a formal control group we apply a quasi‐difference‐in‐differences design where the control group is constructed by extrapolating the pre‐trend of our outcome variable to the post‐period 2012–2013. If there is not much variation over the years in the way mental health care providers treat individual patients in the pre‐period, then it seems reasonable to assume that providers will treat patients in a similar way in the post‐period in absence of a demand shock. We find that a rather flat linear trend provides a good description of the development of our outcome variables in the pre‐period, which indicates that providers' treatment behavior was stable and consistent during the pre‐period.^12^ If we extent this linear trend to the post‐period, we obtain a baseline or counterfactual trend, which represents how providers would have treated their patients if there had not been any policy reforms or demand shocks. A flat trend enhances the credibility of our strategy as it allows us to better identify causal effects in the post‐period. The linear trend is the first important variable in our analysis which is denoted by *baseline*.

The second variable measures the average effect of the demand shock in 2012–2013 and is given by a dummy variable, denoted *shock*, that is, one for the years 2012 and 2013. This variable allows us to measure the average provider responses in the post‐period relative to the baseline.

Our empirical strategy consists of two parts. First, we analyze the major developments in the mental health care sector at an aggregated provider level. Second, we go into more detail by studying the provider behavior at the level of treatment episodes. Both analyses will be done for FFS‐ as well as BUD‐providers. In line with our theoretical framework, we study several dependent variables. At the provider level, the development in the number of treatment episodes and in the total treatment duration is studied. At the level of the treatment episode, we study the change in treatment duration and the change in patient's health outcomes (*DIFGAF*).

### 5.1 Provider level

For the analyses at the provider level we use a fixed effects panel model. Using this model we are able to describe changes in our outcome variables over the years and, specifically, estimate our variables of interest *baseline* and *shock* as described above. We aggregate our data for each mental health care provider at a monthly level. The model is defined as follows:

(13) $$Y_{jym}=c+\beta_{1}baseline+\beta_{2}\text{shock}+\delta_{1}D_{m}+\delta_{2}D_{2008,m}+D_{j}+\epsilon_{jym}$$

where the dependent variable $Y_{jym}$ is the outcome variable for provider j in month m=1,…,12 and year y=2008,…,2013. At the provider level $Y_{jym}$ is either the number of treatment episodes or total treatment duration (in minutes) per provider per month. The baseline trend baseline is a trend variable. $\beta_{1}$ describes the average yearly change in our outcome variable Y over the years 2008 to 2011. The response to the shock in the post‐period is estimated by $\beta_{2}$ and measures the deviation of the outcome variable from the baseline trend as a result of the policy reforms and subsequent demand shock. This provides insight in how providers on average accommodate to the policy reforms and demand shock in 2012–2013.

We include a constant c and month dummies $D_{m}$ to control for within year variation in the outcome variable. $D_{2008,m}$ are dummies for the first 6 months of 2008, which control for the fact that 2008 was the first year that mental health care was reformed from a public to a regulated competition system and providers were still adjusting to this new system.^13^ The error term in the fixed effects panel model is composed of a time‐invariant provider specific effect $D_{j}$ and an idiosyncratic error term $\epsilon_{jym}$. The standard errors are clustered at the provider level.

Model (13) provides a description of the general developments in the sector, as we do not control for case‐mix differences. We will control for those factors in the analyses on treatment episode level below.

### 5.2 Treatment episode level

We zoom in and study provider responses at the treatment episode level to see how the duration for an individual treatment episode has changed. We will estimate the effect on treatment duration without and with controlling for changes in case‐mix. We do this by including patient characteristics as well as attributes specific to the treatment episode. The model with controls is formulated as follows:

(14) $$Y_{ijym}=c+\beta_{1}baseline+\beta_{2}shock+\delta_{1}D_{m}+\delta_{2}D_{2008,m}+D_{j}+\gamma X_{iym}+\epsilon_{ijym}$$

The dependent variable $Y_{ijym}$ represents the dependent variable at the treatment episode level: treatment duration and DIFGAF. Treatment episode i is opened by provider j in month m of year y. In this way we analyze to what extent the treatment duration of a treatment episode changes on average between the pre‐ and post period. As in model (13) we have the same two variables of interest baseline, shock. Also, dummies $D_{m}$ and $D_{2008,m}$ are the same as in model (13).

A difference between models (13) and model (14) is the set of explanatory variables $X_{iym}$ which are specific to treatment episode iym and captures the case‐mix of treatment episode i. $X_{iym}$ includes patient characteristics gender and age, as well as treatment episode characteristics such as the main diagnosis and sub‐diagnosis, the GAF score at the start of the treatment episode, whether a patient stays over night at a mental health institution, and the type of a treatment episode. As there are differences between providers we also include a dummy for each provider, $D_{j}$. Treatment episodes within a provider are likely to be correlated, therefore we cluster the standard errors on the provider level.

The estimates for $\beta_{1}$ and $\beta_{2}$ are in absolute values. In our results section we divide both estimates (and their standard errors) with the predicted counterfactual of the outcome variable in 2012. This allows us to interpret both variables as a percentage change and facilitates the interpretation of the different effects for both types of providers.

## 6 RESULTS

In this section we present the estimations at the provider level (model 13) and treatment episode level (model 14). First, we present the results of both models for the FFS‐providers, and then for the BUD‐providers. At the end of each section we link the results to the hypotheses in our theoretical framework.

### 6.1 FFS‐providers

Table 1 shows the estimation results for FFS‐providers. At the provider level we find, as shown in the first and second column, a positive baseline trend between 2008 and 2011: on average the annual number of treatment episodes increased by 3.8% and the annual total treatment duration by 5.8%. Thus, the production of FFS‐providers has increased over the years both at the extensive margin (i.e., the number of patients) and the intensive margin (i.e., the total number of treatment minutes). Relative to the (increasing) baseline trend, FFS‐providers faced a huge negative demand shock as the number of treatment episodes after the shock declined with 29.9% in 2012 and 2013. Moreover, FFS‐providers lowered their total treatment duration substantially with 21.0% after the shock.

**TABLE 1:** Results for FFS‐providers

	Provider level		Treatment episode level	
	# Treatment	Total	Treatment	*DIFGAF*
	Episodes (%)	Production (%)	Duration (%)	
Baseline ($\beta_{1}$)	3.8	5.8	2.1	−0.003
	(1.2)	(1.3)	(0.4)	(0.006)
	[1.4, 6.2]	[3.2, 8.4]	[1.4, 2.8]	[−0.014, 0.008]
Response 2012–2013 ($\beta_{2}$)	−29.9	−21.0	2.1	0.005
	(3.8)	(3.1)	(0.9)	(0.014)
	[−37.5, −22.4]	[−27.3, 14.7]	[0.4, 3.7]	[−0.023, 0.033]
Controls	Yes	Yes	Yes	Yes
Number of observations	44,908	44,908	252,776	252,776
$R^{2}$	0.061	0.04	0.247	0.304

*Note*: In this table, we present the estimates of the two β's (see Section 5) as a percentage differences from the counterfactual baseline in 2012. Only the last column *DIFGAF* shows absolute differences from baseline. Below the β's, we report the standard errors and 95% confidence intervals. All estimations included all case‐mix controls. At the provider level, we included dummies for year (5), month (11), and providers (740). At the treatment episode level, we included dummies for year (5), month (11), providers (357), main‐diagnoses (18), sub‐diagnoses (121), gender (1), age (98), type of treatment (1), staying overnight (1), and for start GAF (10).

Abbreviation: GAF, Global Assessment of Functioning.

The third and fourth column of Table 1 present the results of our estimations at the treatment episode level. After controlling for case‐mix we find an average increase in treatment duration of 2.1% after the demand shock in 2012–2013.^14^ This result indicates that FFS‐providers treated similar patients on average slightly longer after the demand shock but the increase in treatment duration is small compared to the size of the shock. Finally, column four shows that average treatment outcomes for similar patients did not significantly change over time and after the demand shock.

**FIGURE 5:**
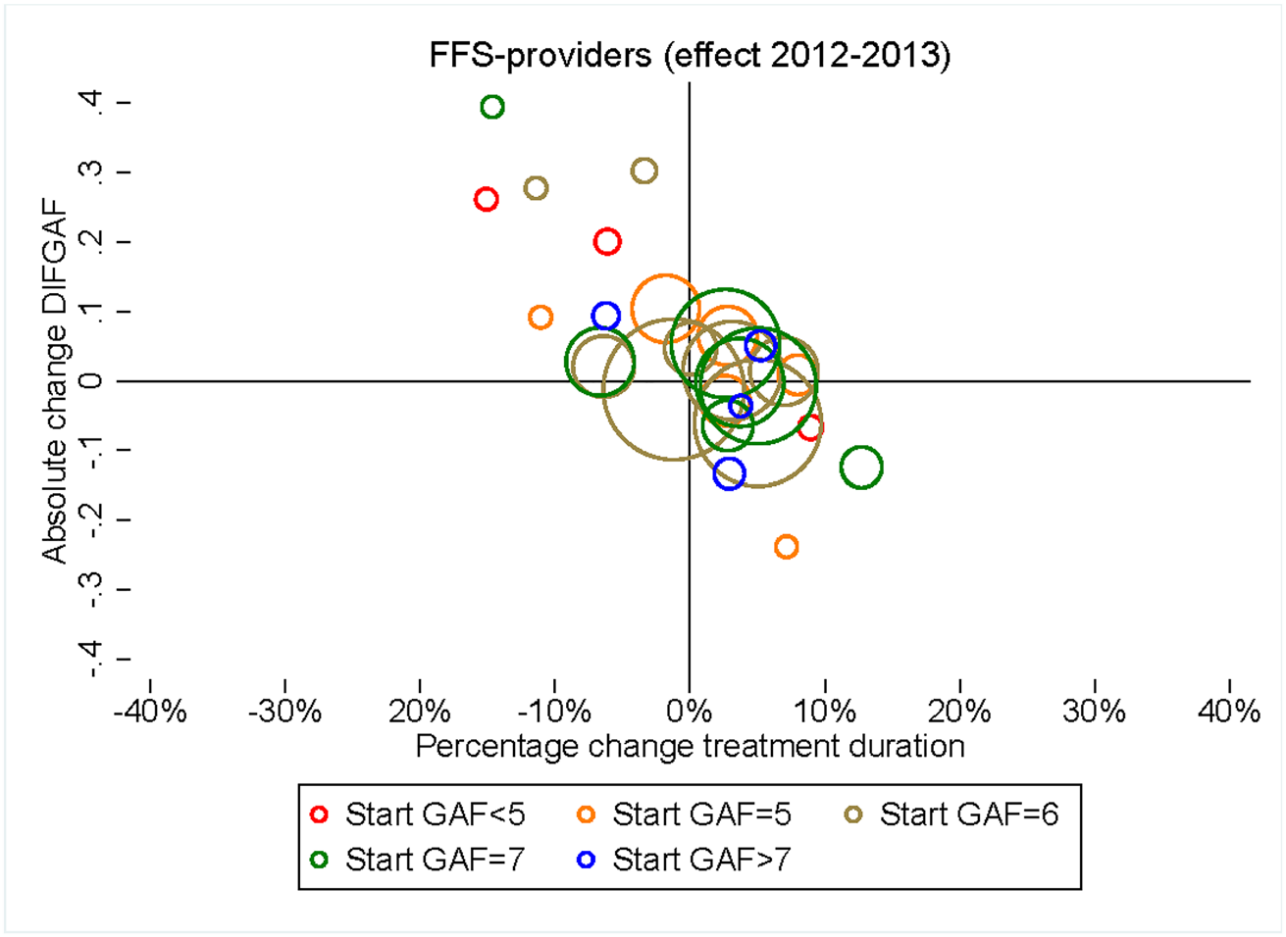
Average responses in 2012–2013 for treatment duration (*x*‐axis) and *DIFGAF* (*y*‐axis) for each combination of diagnosis group (19 groups) and start Global Assessment of Functioning (GAF) level (five groups) when the regression is based on more than 250 observations. This resulted in 27 combinations/circles in the figure. Each of the combinations is represented by the center in a circle, and the size of the circle represents the relative size of the number of observations in a diagnosis‐GAF group

These results do not reveal the variation in effects for different diagnosis groups or start GAF scores. Therefore, we estimate model (14), for every combination of 19 diagnosis groups and five groups of start GAF scores separately. Figure 5 shows the relationship between the responses ($\beta_{2}$) in treatment duration and *DIFGAF* after the demand shock for all combinations of diagnoses and start GAF scores for the regressions with more than 250 observations. The larger the circle, the bigger is the patient group (diagnosis × GAF score). Most large circles are located around the origin suggesting on average no or small changes in treatment duration and *DIFGAF* outcomes.

Important to note here is that for FFS‐providers an increase in treatment duration does not necessarily mean higher reimbursements. Recall that FFS‐providers were paid according to a stepwise payment scheme with treatment duration thresholds at 250, 800, 1,800, 3000 and 6000 min. An increase in treatment duration will result in a higher reimbursement only if accompanied by passing a treatment duration threshold. We therefore run our model (14) also with dependent variable the reimbursement they receive per treatment. Since we have no information about prices we assume in this analyses that providers receive each year the maximum price as determined by the regulator in 2012. Column 1 in Table 2 shows the results. We find a small and insignificant increase of 1.1% in the reimbursement per treatment episode after the shock.

**TABLE 2:** Reimbursement responses for FFS‐providers

	Reimbursement per treatment episode (%)		
	All	Altruistic	Financial
	FFS‐providers	FFS‐providers	FFS‐providers
Baseline ($\beta_{1}$)	1.6	1.4	1.3
	(0.3)	(0.6)	(0.6)
	[1.0, 2.2]	[0.2, 2.6]	[−0.0, 2.5]
Response 2012–2013 ($\beta_{2}$)	1.1	1.1	2.5
	(0.8)	(1.7)	(1.3)
	[−0.4, 2.6]	[−2.3, 4.5]	[0.0, 5.0]
Controls	Yes	Yes	Yes
Number of observations	262,776	62,808	64,730
$R^{2}$	0.230	0.231	0.200

*Note*: In this table, we present both β's (see Section 5) as a percentage differences from the counterfactual baseline in 2012. The notation and control variables are similar as in Table 1.

The response to the shock may differ between FFS‐providers. As we explained in Section 3.2 financially motivated providers, that is, providers with a large $\alpha_{j}^{\text{FFS}}$, may react stronger to a loss in their income than altruistic providers, that is, who have a low $\alpha_{j}^{\text{FFS}}$. As explained in Section 4 we will use the variation around the treatment duration thresholds in Figure 3 to separate both types. The strategy that we follow here is that we classify a provider as financially motivated if she ends many treatment durations at or just after a threshold. We classify a provider as altruistically motivated if she does not react to a threshold. In Appendix D (see Supporting Information S1) we explain in detail how we separate both types from our data. For altruistic FFS‐providers the treatment duration distribution exhibits a smooth trend similar to BUD‐providers in Figure 3.

The second and third column in Table 2 show the estimated reimbursement responses for both types of providers. Again, the effects are small. We find a small and insignificant increase of 1.1% for altruistically motivated providers after the shock. This is as expected. Altruistic providers do not respond to the treatment duration thresholds so we also do not expect them to change treatment duration, to obtain a higher reimbursement, after the demand shock. The effect for the financially motivated providers is with 2.5% somewhat larger but the increase is still relatively small compared to the large demand shock.

An important reason for not finding stronger income effects for financially motivated providers is likely to be related to the discontinuities in the stepwise fee‐for‐service system. To obtain a higher reimbursement would imply that providers have to increase treatment duration beyond a next threshold. This would imply a huge change in treatment behavior because they ended most treatments already on or just after a threshold before the shock. For example, a financially motivated provider ending a treatment at 800 min before the demand shock has to more than double treatment duration to at least 1800 min to obtain a higher reimbursement. This suggests that although financially motivated providers put a low weight, that is, low $\alpha_{j}^{\text{FFS}}$, to patient benefits it is still positive in their utility function.

To conclude, and to answer our first two hypotheses from Section 3.2, we do not find evidence of an income effect for FFS‐providers as we find only a small and insignificant increase in treatment duration after the demand shock. The treatment duration thresholds in the stepwise fee‐for‐service payment function seem to have prevented financially motivated providers to treat patients longer. As a result we find no changes in *DIFGAF* outcomes before and after the shock. Thus, the demand shock resulted in a loss in production of about 20% and corresponding income for FFS‐providers.

### 6.2 BUD‐providers

Table 3 shows the results for BUD‐providers. The baseline trend at the provider level in the first two columns is fairly constant between 2008 and 2011, as $\beta_{1}$ is small and insignificant. In line with Figure 1 we find a large decline in the average number of treatment episodes by 19.3% in 2012–2013. The second column shows that total production at the provider level, measured in total treatment minutes, on average drops by 9.6% in 2012–2013. The change in total production is smaller than the change in the number of treatment episodes which suggests an increase in average treatment duration in 2012–2013.

**TABLE 3:** Results for BUD‐providers

	Provider level		Treatment episode level	
	# Treatment	Total	Treatment	
	Episodes (%)	Production (%)	Duration (%)	*DIFGAF*
Baseline ($\beta_{1}$)	−0.8	1.0	1.2	0.009
	(0.9)	(0.8)	(0.4)	(0.006)
	[−2.6,1.0]	[−0.6,2.7]	[0.6,2.0]	[−0.003,0.021]
Response 2012–2013 ($\beta_{2}$)	−19.3	−9.6	7.9	−0.006
	(3.9)	(2.6)	(1.0)	(0.010)
	[−27.0,−11.6]	[−14.7,−4.6]	[5.9,10.0]	[−0.025,0.013]
Controls	Yes	Yes	Yes	Yes
Number of observations	23,635	23,635	3,892,093	3,892,093
$R^{2}$	0.004	0.002	0.227	0.189

*Note*: In this table, we present the estimates of the two β's (see Section 5) as a percentage differences from baseline. Only the last column *DIFGAF* shows absolute differences from baseline. Below the β's, we report the standard errors and 95% confidence intervals. All estimations included all case‐mix controls. At the provider level, we included dummies for year (5), month (11), and providers (357). At the treatment episode level, we included dummies for year (5), month (11), providers (357), main‐diagnoses (18), sub‐diagnoses (121), gender (1), age (98), type of treatment (1), staying overnight (1), and for start GAF (10).

Abbreviation: GAF, Global Assessment of Functioning.

After controlling for case‐mix, the third column shows that BUD‐providers have increased their treatment duration substantially after the demand shock. Indeed, we find a large significant increase in average treatment durations by 7.9% compared to baseline at the treatment episode level. Controlling for case‐mix is important as without controls we find larger increases in average treatment duration of 12.2%.^15^ Changes in the case‐mix however do not fully explain the increase in treatment duration. The last column of Table 3 shows that our outcome measure *DIFGAF* did not change before and after the demand shock, $\beta_{2}$ is both negative and insignificant at the 5% level, indicating that longer treatment durations have on average not improved patient outcomes, as measured by GAF‐scores.

**FIGURE 6:**
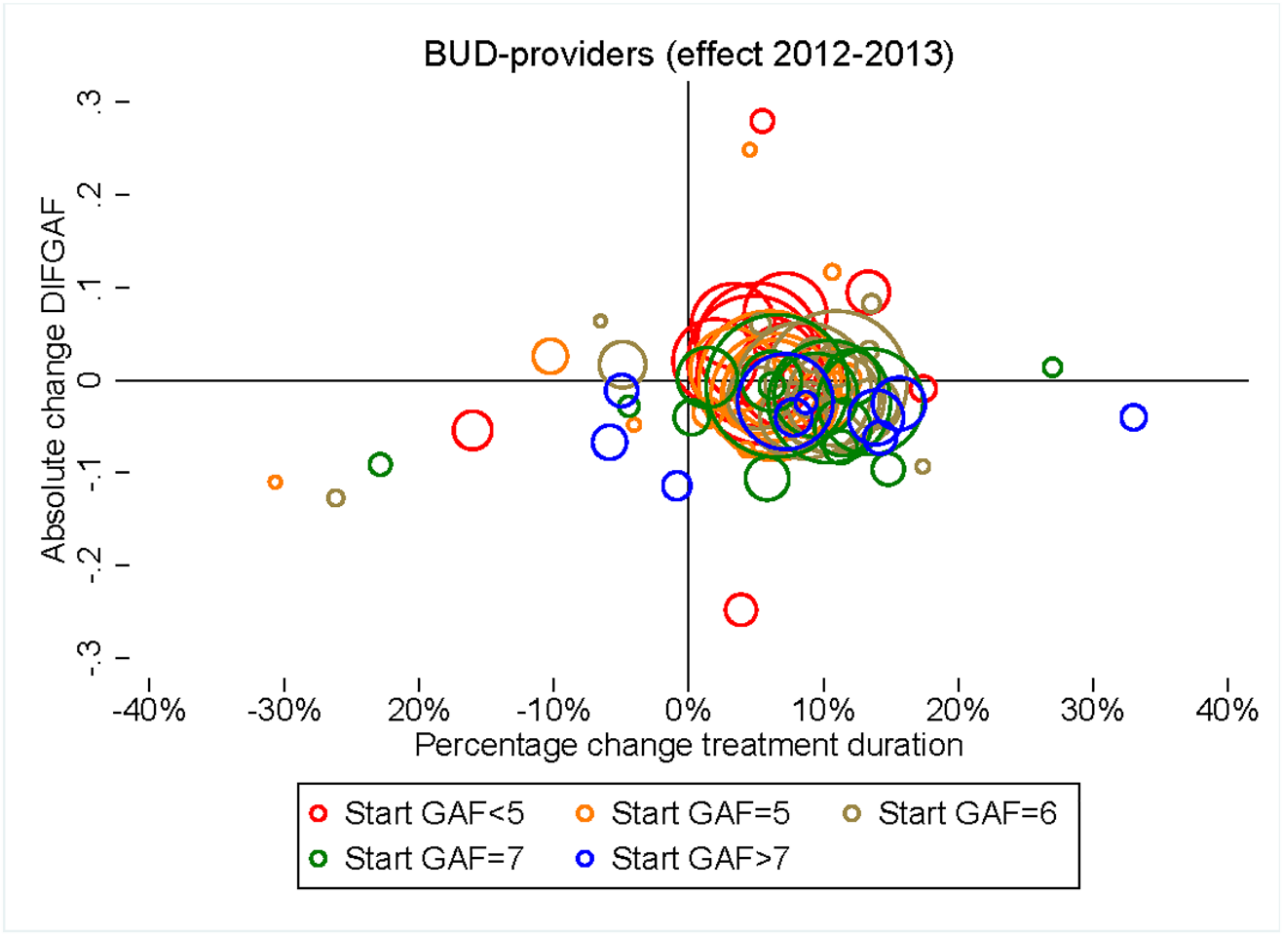
Average responses in 2012–2013 for treatment duration (*x*‐axis) and *DIFGAF* (*y*‐axis) for each combination of diagnosis group (19 groups) and start Global Assessment of Functioning (GAF) level (five groups) when the regression is based on more than 250 observations. This resulted in 75 combinations/circles in the figure. Each of the combinations is represented by the center in a circle, and the size of the circle represents the relative size of the number of observations in a diagnosis‐GAF group

Figure 6 shows at the treatment episode level the relationship between the change in treatment duration and *DIFGAF* outcomes for 2012–2013. The figure shows that treatment duration (on the horizontal axis) increases for the vast majority of the treatment episodes while the changes in GAF‐scores (on the vertical axis) all lie around zero. Especially, we observe no large circles (representing many observations) at the left of zero indicating that treatment duration increased for all combinations with a large group of patients.

The results in Figure 6 allow us to discuss some important points. First, we find increases in treatment duration also for severe patients (low GAF scores) for which the number of patients did not decline in the post‐period. Second, we assumed in our *DIFGAF* estimations for the full model that improvement in health is independent of severity: a difference of 1 in *DIFGAF* is the same whether the start GAF‐score is 3 or 7. In the estimations we relax this assumption as we use the same (group of) start GAF‐scores. A study of the results indicates that improvements and deteriorations in GAF‐scores are quite randomly distributed in Figure 6, suggesting also no clear improvement in outcomes.

Our theoretical model yields two possible explanations for longer treatment durations: professional uncertainty and income effects. The professional uncertainty effect implies that BUD‐providers have experienced some form of implicit or explicit rationing before the shock. After the shock, when more capacity became available, providers treated their patients longer because they had more time available per patient and they believed it would benefit these patients. Note that professional uncertainty is not a financial effect but driven by the beliefs of providers to treat patients in the best possible way. Empirically, the professional uncertainty and income effects are extremely difficult to disentangle, but we believe that in this practical case professional uncertainty plays a more important role, at least in the short term, than income and price effects. First, most employees in these large institutions have long term contract, fixed working times and a salary, thus treating patients longer has no direct consequences for their own income. Second, to solve our theoretical model we had to assume at the organizational level a hard budget constraint while in practice budget constraints are soft. In the case of a hard budget constraint budget cuts should be 15% or larger, that is, due to fall in prices and number of treatments. However, as we explain in Appendix E (see Supporting Information S1), large budget cuts did not occur and also the number of employees in the market remained relatively stable. Third, if income effects would play an important role then this would suggest a positive correlation between the size of the demand shock and the size of the response. However, in Appendix E (see Supporting Information S1) we find no evidence for this correlation.

To conclude, and to answer our second hypothesis of Section 3.3, we find that BUD‐providers responded to the shock by increasing average treatment duration for similar patients by about 8%. This suggests that professional uncertainty and/or income effects dominate possible substitution effects. Additional evidence suggest that professional uncertainty is a more likely explanation than income effects. Our answer to the third hypothesis in Section 3.4 is that we do not find an improvement in *DIFGAF* outcomes which yields suggestive evidence for overtreatment.

### 6.3 Robustness analyses

To test if our results are not driven by other mechanisms, we have performed several additional analyses. The results are listed in Appendix F (see Supporting Information S1). Here we summarize the main findings.

Overtreatment is only present if longer treatment durations are related to direct treatment time, that is, face‐to‐face time with patients, and not to indirect time, such as administrative tasks. If we perform the same estimations with direct treatment time we find similar responses. This suggests that providers increased their direct and indirect treatment time in the same proportion after the shock.

There may have been anticipation effects to the demand shock just before and after. We test this anticipation effect by estimating the models leaving out all observations from July 2011 until June 2012. The results are again comparable with the main results in our study, albeit the responses become somewhat stronger for BUD‐providers.

We test whether recoding of diagnoses influences our results by running our regressions for the providers that had the fewest diagnoses for “adjustment” and “V‐codes” in the pre‐period. Provider responses are now somewhat smaller for BUD‐providers which is likely related to the somewhat smaller size of the demand shock for these diagnoses.

If treatment durations are extended this means that our end GAF scores are measured at an earlier or later point in time in the post‐period. This could influence our treatment outcomes if patients start a treatment when they are in worse condition and mental health outcomes may tend to mean revert irrespective of a treatment. Therefore we run the same regressions with total number of days a treatment lasted. We find a response of 3.4% for BUD‐providers after the shock. The effect is smaller than the treatment duration effect which can be explained by the fact that providers received more capacity and thus could plan recurring visits sooner. The effect is still positive which could support that the mean revert effect was stronger after than before the shock which would bias our *DIFGAF* results upwards. Since we find no change in outcome the mean revert effect is likely to be small.

## 7 CONCLUSION AND DISCUSSION

This paper studies how Dutch mental health care providers responded to a large drop in the number of patients, both in terms of treatment duration and treatment outcomes. We compare self‐employed providers (FFS‐providers) who are paid according to a discontinuous stepwise fee‐for‐service scheme with mental health care institutions paid by an annual budget (BUD‐providers). Our main result is that both types of providers responded to the demand shock differently and that this is the result of their payment system.

FFS‐providers hardly changed their patients' average treatment duration after the demand shock. In theory, FFS‐providers may have a financial incentive to increase their income by prolonging treatment duration after a negative income shock. Firstly because the loss in income after the negative demand shock does not allow them to run a profitable business. Secondly, financially motivated providers may want to recoup some of their loss in income. Empirically we find that both altruistically and financially motivated FFS‐providers hardly respond to the shock. In case of altruistically motivated providers this is because they weigh patient benefits more than their own income in their utility function. However, financially motivated providers did not respond to the shock either. The reason is that FFS‐providers exploited the stepwise fee‐for‐service payment system already before the demand shock, and income could only be further increased via an unreasonably large increase in treatment duration.

BUD‐providers increased their average treatment duration by 7.9% after the demand shock. Our theoretical model provides two possible reasons for this: an income effect (similar as with FFS‐providers) and a professional uncertainty effect. Both of these effects dominate the substitution effect. The professional uncertainty effect implies that BUD‐providers have experienced some form of implicit or explicit rationing before the shock. After the shock, when more capacity becomes available, providers treat their patients longer because they have more time and capacity per patient and they believe it would benefit these patients. Professional uncertainty is not a financial effect but it is driven by provider beliefs about treatment outcomes. Empirically, the professional uncertainty and income effects are difficult to disentangle. However, we argue that professional uncertainty plays a more important role than income and price effects as these are likely to be small (at least in the short term). Firstly because the income of most employees in these large institutions do not directly depend on treatment duration. Secondly because budget constraints are soft in practice. A hard budget constraint would imply budget cuts of 15% or larger due to the fall in tariffs and the number of treatments, but such large budget cuts are unlikely. We conclude that BUD‐providers weigh patient benefits more than income in their utility function. Moreover, if income effects would play an important role then this would suggest a positive correlation between the size of the demand shock and the size of the response, which we do not find empirically.

Our study provides suggestive evidence for overtreatment as longer treatments did not result in an improvement in GAF‐scores (i.e. General Assessment of Functioning). This may be because ex‐ante provider beliefs on how to treat a patient optimally differ from actual outcomes. The mental healthcare is known for its supply sensitive treatments (Koekkoek et al., 2019). Another reason for this finding is that we do not measure all dimensions of quality and that quality may have improved at other unobservable dimensions.

The study has several limitations. A first limitation is that we lack a control group and had to apply a quasi‐difference‐in differences approach. However, the rather flat trend of the outcome variables in the pre‐period combined with the large size of the shock in the post‐period enhances the credibility of our strategy. A second limitation is that we have only one outcome indicator. The GAF score has great advantages as the indicator is measured before and after each treatment by the (same) provider. Despite critique on the GAF (Vatnaland et al., 2007), it is known as a reliable indicator (Hilsenroth et al., 2000), especially when comparing groups of patients (Jones et al., 1995; Soderberg et al., 2005). Alternative outcome measures, such as Routine Outcome Monitoring, were not available for the years that we investigated. A third limitation might be that we do not have enough information to control sufficiently for case‐mix in our regressions. However, we believe that these effects are small because the responses for FFS‐providers almost completely disappear in our current regressions after controlling for case‐mix.

This study also provides some lessons for policy and the design of payment models. Restrictions to payment models, such as thresholds in a fee‐for‐service scheme or a budget restriction, may be useful in the case of supply sensitive treatments as they may prevent overtreatment by financially motivated providers. Our study shows that restricting provider capacity may also prevent overtreatment of providers with biased beliefs about treatment possibilities. In the longer run payment models should be based on treatment outcomes. However, as long as there is insufficient information about the quality of treatments, smart restrictions can be useful tools. The stepwise fee‐for‐service system suffers from the strong unintended responses at treatment duration threshold by financially motivated providers. However, an advantage is that these unintended responses are easy to monitor which allows insurers to control providers. Also, in a stepwise system there is not always an incentive to induce demand at the margin. Moreover, as our study shows, the stepwise fee‐for‐service system might be more immune against exogenous shocks as the thresholds seem to prevent large changes in treatment duration.

## CONFLICT OF INTEREST

The authors declare no conflict of interest.

## Supporting information

Supporting Information S1

## Data Availability

Research data are not shared.

## ENDNOTES

^1^
See also Lambregts and van Vliet (2018) and Ravesteijn et al. (2017).

^2^
Previous researchers have used (implicit) outcome measures, such as mortality rates (such as Clemens & Gottlieb, 2014), survival rates (Jacobson et al., 2017), treatment choices (Gruber & Owings, 1996), or a variety of hospital conditions (Currie et al., 2016; Doyle et al., 2015, 2017).

^3^
Patients with mild mental health conditions can receive treatment in primary mental health care.

^4^
DBC stands for diagnosebehandelcombinatie in Dutch.

^5^
The DBC has strong similarities with a Diagnostic Related Group that is used in many other countries. See Westerdijk et al. (2012).

^6^
An example of a DBC is “Depression, 250 to 800 min of treatment, no medication.”

^7^
In general FFS‐providers negotiate with insurers a percentage of the maximum tariff. We have no information about these negotiated percentages but most of these percentages are between 75% and 100% of the maximum tariff (Dutch Healthcare Authority, 2013).

^8^
Patients with adjustment disorders suffer from stressful events and patients with V‐code disorders have relational or occupational problems. Before the demand shock, adjustment disorders covered about 10% and V‐codes about 7% of all curative mental health services.

^9^
The reason for the abolishment of the deductible in 2013 were related to budgetary windfalls in 2013 and, presumably, the unexpected strong response by patients to the deductible which attracted a lot of media attention.

^10^
See Owen et al. (2016) for some evidence of this assumption in mental health care.

^11^
For a detailed description of the Global Assessment of Functioning score, see the DSM‐V handbook of American Psychiatric Association (2000).

^12^
For example, adding a quadratic or logarithmic term to the linear term in the pre‐period regressions at the treatment episode level turned out to be insignificant in all cases.

^13^
We tested this empirically. The adjustment effects of the 2008 reforms disappeared after about 6 months, and adding more monthly dummies did not alter our results.

^14^
Note that controlling for case‐mix is important. Without controls we find an increase of 8.0%. These results are available upon request.

^15^
These results are available upon request.
