# Supplementary material for: Payment schemes and treatment responses after a demand shock in mental health care
Source: Health Econ. 2021 Sep 7;30(12):2956–73. doi: 10.1002/hec.4417 (PMC9291998; doi:10.1002/hec.4417)
Supplement: Supplementary file 1 — Supporting Information S1 [file HEC-30-2956-s001.pdf]

## Appendix A

Figure 1: Example of stepwise fee-for-service payment scheme for FFS-providers

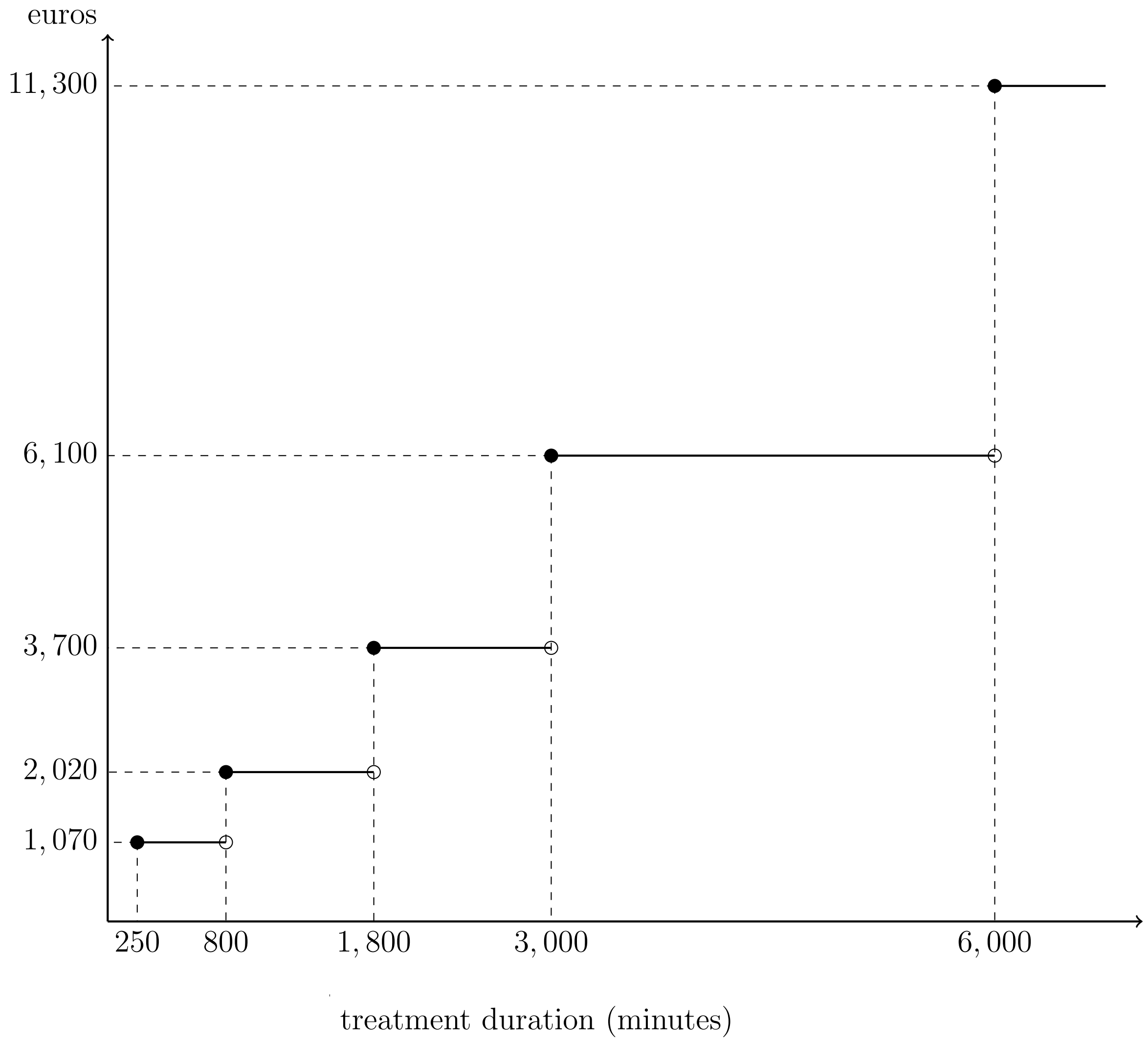


Note: The stepwise fee-for-service function in this example applies to the diagnosis schizophrenia. The treatment duration thresholds at x-axis are the same for all diagnoses. The tariffs at the y-axis are the maximum tariffs in 2011. The tariffs are rounded off and differ only slightly across diagnoses.

## Appendix B: Sample selection

For the analyses the data were cleaned in several steps. Firstly, treatment episodes which included missing values and outliers were removed. Next, we used only the majority of treatment episodes that were labeled as a "regular" or "continued" treatment and removed other less common treatment episodes. In the case a regular treatment lasts longer than 365 days, a provider has to start a new treatment episode for administrative reasons labeled "continued treatment". Treatment episodes with a very long treatment duration, that is, over 8,000 minutes, were also removed from the data as they are uncommon, may be outliers, and may refer to very specialized treatments. As we want to study treatment duration responses of incumbent providers that were on the market before and after the shock, we constructed a balanced data set and selected only providers that were all years on the market. To obtain this final sample roughly 30% of the treatment episodes in the raw data were removed.

Table 1: Overview of data cleaning and sample selections

| | Raw sample | Selection 1 (cleaning) | Selection 2 (selecting type of treatment episode) | Selection 3 (selecting durations) | Final sample (balancing) |
|---|---|---|---|---|---|
| Total | | | | | |
| - number of providers | 2,640 | 2,593 | 2,592 | 2,592 | 1,097 |
| - number of treatment episodes | 5,948,857 | 5,297,349 | 4,787,621 | 4,673,431 | 4,146,555 |
| FFS-providers | | | | | |
| - number of providers | 1,738 | 1,716 | 1,716 | 1,716 | 740 |
| - number of treatment episodes | 432,860 | 392,275 | 383,910 | 383,348 | 253,261 |
| BUD-providers | | | | | |
| - number of providers | 902 | 877 | 876 | 876 | 357 |
| - number of treatment episodes | 5,515,997 | 4,905,074 | 4,403,711 | 4,290,083 | 3,893,294 |

Table 1 shows the various steps in our data cleaning and sample selection process. The first column refers to all observations in our raw sample for the years 2008-2013. Column Selection 1 refers to the first cleaning step where we removed providers and treatment episodes with missing values and outliers. Column Selection 2 refers to the selection of all "regular" and "continued" treatments. To keep our sample as homogenous as possible we removed treatment episodes that were mainly short and less common such as one-time (urgent) consults, intercollegial consultations or second opinions, and acute admissions of patients for intensive treatment. Column Selection 3 refers to the selection of all treatments with a duration less than 8,000 minutes. Column Selection 4 refers to the selection of all incumbent providers that were on the market

during all years 2008-2013. To obtain a panel dataset we balanced on yearly not monthly level. It is therefore possible that a provider does not record treatment episodes in a particular month between 2008 and 2013.

## Appendix C: Descriptive statistics

Table 2: Descriptive statistics FFS-providers

| | 2008 | 2009 | 2010 | 2011 | 2012 | 2013 |
|---|---|---|---|---|---|---|
| Number of non-budgeted providers | 740 | 740 | 740 | 740 | 740 | 740 |
| Number of treatment episodes | 40,655 | 46,733 | 47,545 | 48,117 | 35,851 | 34,360 |
| - Mood | 8,565 | 9,645 | 9,504 | 10,659 | 10,159 | 12,254 |
| - Anxiety | 6,455 | 7,465 | 7,772 | 9,273 | 8,682 | 9,962 |
| - Personality | 5,086 | 5,651 | 5,607 | 5,921 | 5,870 | 6,140 |
| - Adjustment | 6,614 | 7,596 | 7,657 | 4,529 | 199 | 20 |
| - V-codes | 10,037 | 11,547 | 11,736 | 11,468 | 5,145 | 116 |
| - Start GAF $< 5$ | 611 | 568 | 698 | 811 | 701 | 596 |
| - Start GAF $= 5$ | 4,302 | 4,768 | 4,418 | 5,036 | 4,529 | 5,174 |
| - Start GAF $= 6$ | 20,550 | 22,701 | 22,560 | 23,179 | 17,788 | 17,457 |
| - Start GAF $= 7$ | 12,617 | 15,125 | 15,723 | 15,016 | 10,413 | 9,073 |
| - Start GAF $> 7$ | 2,575 | 3,571 | 4,146 | 4,075 | 2,420 | 2,060 |
| Number of treatment episodes per patient | 1.06 | 1.08 | 1.12 | 1.14 | 1.10 | 1.09 |
| | | | | | | |
| Average treatment duration | 902 | 927 | 933 | 995 | 1,138 | 1,138 |
| - Start GAF $< 5$ | 1,125 | 1,100 | 946 | 1,062 | 1,158 | 1,227 |
| - Start GAF $= 5$ | 1,092 | 1,090 | 1,169 | 1,264 | 1,408 | 1,408 |
| - Start GAF $= 6$ | 958 | 999 | 1,011 | 1,067 | 1,199 | 1,174 |
| - Start GAF $= 7$ | 798 | 839 | 837 | 881 | 1,002 | 986 |
| - Start GAF $> 7$ | 585 | 597 | 619 | 657 | 765 | 790 |
| Average direct time | 632 | 642 | 645 | 691 | 793 | 791 |
| Average indirect time | 269 | 283 | 286 | 300 | 340 | 335 |
| | | | | | | |
| Average Start GAF | 6.29 | 6.35 | 6.38 | 6.34 | 6.25 | 6.19 |
| Average DIFGAF | 0.887 | 0.856 | 0.818 | 0.878 | 0.846 | 0.891 |
| | | | | | | |
| Average age patient | 38.78 | 38.72 | 39.07 | 39.05 | 38.25 | 38.76 |
| Share females | 64.30% | 64.35% | 64.59% | 64.38% | 63.09% | 64.72% |
| Type of care | | | | | | |
| - Share regular treatment | 79.22% | 71.93 % | 70.23% | 70.35% | 69.37% | 70.08% |
| - Share continued treatment | 20.78% | 28.07% | 29.77% | 29.65% | 30.63% | 29.92% |

Notes: Treatment duration, average direct and indirect time are specified in minutes. Average DIFGAF is Total DIFGAF divided by the total number of treatment episodes in a given year.

Table 3: Descriptive statistics BUD-providers

| | 2008 | 2009 | 2010 | 2011 | 2012 | 2013 |
|---|---|---|---|---|---|---|
| Number of budgeted providers | 357 | 357 | 357 | 357 | 357 | 357 |
| Number of treatment episodes | 687,854 | 720,420 | 702,249 | 681,507 | 548,372 | 552,892 |
| - Mood | 136,533 | 134,075 | 129,286 | 137,379 | 125,265 | 132,486 |
| - Anxiety | 74,846 | 78,578 | 76,967 | 82,299 | 74,288 | 80,983 |
| - Personality | 49,541 | 51,205 | 51,578 | 55,349 | 51,577 | 58,956 |
| - Adjustment | 67,621 | 73,024 | 69,291 | 44,755 | 2,558 | 832 |
| - V-codes | 71,779 | 72,621 | 66,555 | 58,767 | 26,773 | 1,986 |
| - Start GAF $<5$ | 72,748 | 67,769 | 65,961 | 60,850 | 52,901 | 56,948 |
| - Start GAF $=5$ | 155,817 | 174,607 | 173,890 | 176,044 | 157,972 | 170,640 |
| - Start GAF $=6$ | 279,689 | 304,823 | 300,321 | 294,811 | 236,632 | 234,802 |
| - Start GAF $=7$ | 137,691 | 136,788 | 128,814 | 120,031 | 81,730 | 73,861 |
| - Start GAF $>7$ | 41,909 | 36,433 | 33,263 | 29,771 | 19,137 | 16,641 |
| Number of treatment episodes per patient | 1.08 | 1.06 | 1.07 | 1.08 | 1.06 | 1.07 |
| | | | | | | |
| Average treatment duration | 1,169 | 1,215 | 1,232 | 1,251 | 1,427 | 1,483 |
| - Start GAF $<5$ | 1,524 | 1,704 | 1,744 | 1,726 | 1,868 | 1,919 |
| - Start GAF $=5$ | 1,433 | 1,481 | 1,514 | 1,536 | 1,675 | 1,742 |
| - Start GAF $=6$ | 1,173 | 1,183 | 1,181 | 1,202 | 1,367 | 1,399 |
| - Start GAF $=7$ | 850 | 865 | 863 | 869 | 1,007 | 997 |
| - Start GAF $>7$ | 587 | 617 | 633 | 629 | 713 | 694 |
| Average direct time | 660 | 664 | 671 | 693 | 785 | 796 |
| Average indirect time | 471 | 512 | 521 | 511 | 560 | 569 |
| | | | | | | |
| Average Start GAF | 5.77 | 5.79 | 5.77 | 5.76 | 5.67 | 5.62 |
| Average DIFGAF | 0.2330 | 0.2157 | 0.2164 | 0.2709 | 0.2639 | 0.2613 |
| | | | | | | |
| Average age patient | 38.98 | 38.51 | 38.31 | 38.16 | 37.10 | 37.96 |
| Share females | 51.86% | 51.41% | 51.24% | 51.55% | 50.11% | 50.53% |
| Type of care | | | | | | |
| - Share regular treatment | 53.60% | 54.67% | 54.03% | 53.70% | 51.85% | 54.63% |
| - Share continued treatment | 46.40% | 45.33% | 45.97% | 46.30% | 48.15% | 45.37% |

Notes: Treatment duration, average direct and indirect time are specified in minutes. Average DIFGAF is Total DIFGAF divided by the total number of treatment episodes in a given year.

## Appendix D: Separating financially and altruistically motivated providers

Figure ?? shows that FFS-providers respond to the treatment duration thresholds. This suggests that FFS-providers are sensitive to financial incentives. We use these responses to separate financially from altruistically motivated providers. We classify providers as altruistically motivated if they put less weight to their own income compared to patient benefit, thus these providers do not respond to the thresholds. Financially motivated put more weight on their own income and respond to the tresholds by ending their treatment duration more often at or just after a threshold.

To separate both types of providers we follow Douven et al. (2019). We first measure the distance measure between each treatment duration $x_{ijt}$, for treatment episode $i$, provider $j$ and year $t$ to the last past treatment duration threshold $k_{l-1}$, which are given in Figure 1:

$$distance_{ijt} = \frac{k_l - x_{ijt}}{k_l - k_{l-1}} \in [0, 1]. \tag{1}$$

A treatment $x_{ijt}$ that is equal to the threshold $k_{l-1}$ obtains a normalized difference score of one, while a treatment ended just before the subsequent threshold $k_l$ gets a normalized difference score of zero. We translate this difference per treatment to an overall altruism score provider $j$ at time $t$ by calculating the average squared difference over all treatments by a provider during 2008-2011:

$$altruism_j = 1 - \frac{\sum_{i=1}^{N_j} distance_{ij}^2}{N_j} \in [0, 1], \tag{2}$$

where $N_j$ are the number of treatment episodes of a provider during 2008-2011. We use the squared distance to emphasize that we can only classify providers who end their treatment relatively often after a threshold as financially motivated. $altruism_j$=0 implies that a provider is strongly financially motivated and ends always at, $k_{l-1}$ while a score of 1 implies that provider $j$ always ends a treatment just before threshold $k_l$. Next we split up our treatment episodes in four different quartiles using $altruism_j$. We define the group of providers in the first quartile as financially motivated and in the fourth quartile as altruistically motivated.

Figure 2 shows the distribution of treatment duration of both groups for 2008 tp 2011 (solid line) and 2012 to 2013 (dashed line). It is clear from both figures that financially motivated providers are strongly responsive to the thresholds while the altruistically motivated providers are not. For example, if we translate these responses to earnings then financially motivated providers earn on average about 20 euros more per hour than altruistically motivated providers, i.e. 128 versus 108 euros per hour. Moreover, Douven et al. (2019) show that during 2008 and 2010 altruistic providers had significantly better treatment outcomes, i.e. changes in GAF-scores, than financially motivated providers.

Figure 2: Distribution of treatment duration

Altruistically motivated FFS providers

Financially motivated FFS-providers

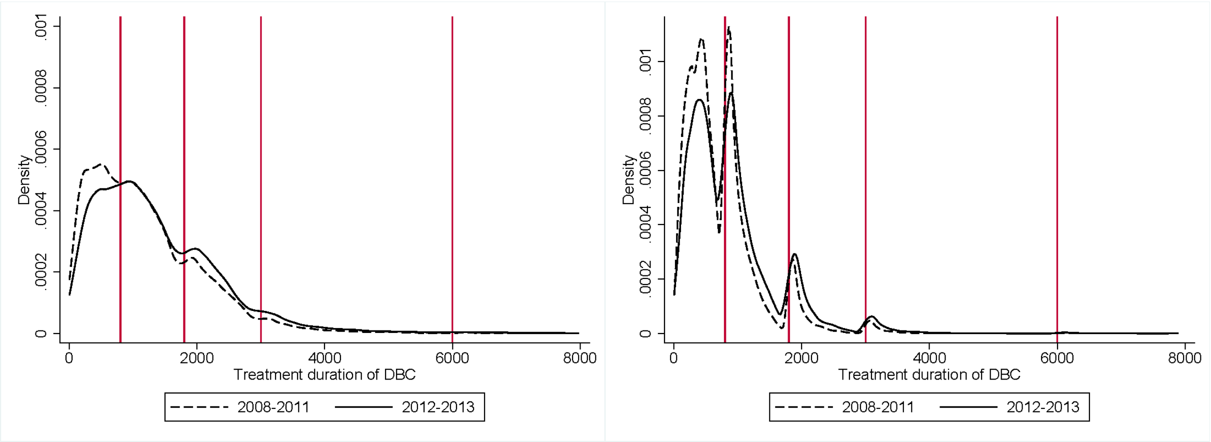

## Appendix E: Professional uncertainty effect versus income effect

In our empirical analyses we find an increase in average treatment duration of 7.9% for BUD-providers. In our theoretical section we relate this increase to a professional uncertainty effect and income effect. In this appendix we argue that the professional uncertainty effect is more likely an explanation for the increase than the income effect.

First, most employees in these large institutions have long term contract, fixed working times and a salary, thus treating patients longer has no direct consequences for their own income.

Second, an income effect would imply budget cuts of 15% or larger, i.e. due to fall in prices and number of patients. However, such large budget cuts did not happen. While the number of patients declined substantially by about 20% (see Table 4) total spending on Dutch secondary mental health care remained relatively stable in 2012-2013. Table 4 shows that the budget declined by 0.2 billion euros between 2011 and 2012, which roughly corresponds to the 5.5% cut in maximum price tariffs for FFS-providers and a 5.5% reduction by the government in the annual budgets for BUD-providers. The government did not incorporate further ex-ante budget cuts because its intention was to limit growth in mental health care spending.[1] This argument is reinforced by the fact that the large negative demand shock was unforeseen by players in the market. Moreover, there were also few incentives for representative regional insurers to cut budgets further as they run almost no financial risk. Larger budget cuts could put many BUD-providers, many of them having considerable fixed costs, into serious problems. These arguments suggests that BUD-providers put a relative higher weight to patient benefits than to income in their utility function.

Table 4: Number of patients, total spending and employees in Dutch secondary mental health care sector

| Year | 2009 | 2010 | 2011 | 2012 | 2013 |
|---|---|---|---|---|---|
| Number of patients (x 1,000) | 767 | 750 | 730 | 584 | 587 |
| Spending (x 1 billion euros) | 3.8 | 4.0 | 4.3 | 4.1 | 4.0 |
| Number of employees (x 1,000) | 87 | 87 | 89 | 89 | 89 |

Notes: The number of patients corresponds to the unique patients in our balanced panel that we study in Section ??. Total spending figures are retrieved from Dutch Healthcare Authority (2014, 2016) and the number of employees from www.azwinfo.nl (retrieved at April, 23, 2019).

The number of employees and full-time employees in the mental health care sector remained

[1] From a budgetary perspective the policy measures turned out to be successful for the government as the growth of secondary mental health care spending had stopped in 2012-2013.

relatively stable during 2012 and 2013, which indicates that the large decline in the number of patients did not result in a corresponding decline in the number of employees. Table 4 shows that the number of workers in the total mental health care sector increased during 2009 to 2011 up to 89,000, but remained constant between 2011 and 2013.[2] This suggests that the sudden decline in the number of patients during 2012 and 2013 has increased the relative capacity of providers to treat patients.

Table 5: Treatment duration responses of BUD-providers ranked according to the size of the shock.

| | *Treatment duration responses BUD-providers (%)* | |
|---|---|---|
| | First Quartile (large shock) | Fourth quartile (small shock) |
| Baseline ($\beta_1$) | 1.8 | 0.9 |
| | (0.6) | (0.7) |
| | $[0.5, 3.1]$ | $[-0.6, 2.4]$ |
| Response 2012-2013 ($\beta_2$) | 6.9 | 7.5 |
| | (1.8) | (1.5) |
| | $[3.8, 10.5]$ | $[4.7, 10.5]$ |
| Controls | yes | yes |
| Number of observations | 977,422 | 1,009,564 |
| $R^2$ | 0.236 | 0.243 |

Notes: Quartile 1 corresponds to 133 BUD-providers that faced the largest negative demand shock (29% decline in the number of treastment episodes or more). Quartile 1 includes 133 BUD-providers and faced the smallest shock (14% decline or less). The notation and control variables are similar as in Table **??**

Third, if income effects would play an important role then this would suggest a positive correlation between the size of the demand shock and the size of the response. The income effect implies that providers try to recoup some of their loss in income by treating patients longer. However, a positive correlation may not be present in the case of professional uncertainty. For example, in the case of professional uncertainty providers that observe a large negative demand shock may decide not to treat patients longer because they believe that treatment duration was already optimal in the pre-period and longer treatments in the post-period are not beneficial for their patients. Thus, if we would not find a (strong) positive correlation then this would be sooner an indication of a professional uncertainty effect than an income effect. For BUD-providers we have no exogenous variation related to discontinuities in the payment system as

[2]This holds also for full-time employees which increased during 2009-2011 from 71,000 to 73,000 but remained relatively stable with 72,000 and 73,000 in 2012 and 2013. Only in 2014 there was a drop in employment to 71,000.

for FFS-providers, however we can try to shed some more light on the two explanations by following the strategy of Yip (1998). She estimated provider responses after a large reduction in Medicare prices after a reform. She found evidence of an income effect hypothesis by showing that providers with the largest losses in their income had also the largest volume responses to recoup this income. We perform a similar exercise and group BUD-providers in four quartiles. The first quartile consists of 133 providers that faced the largest reduction in treatment episodes (29% or more) in the post-period compared to the pre-period. The fourth quartile consists of 133 providers with the smallest reduction (14% or less). If the income effect would play a role then we would expect a positive correlation between prolonging treatment duration and the magnitude of the demand shock after the policy reform. The other explanation, prior rationing combined with professional uncertainty, is less likely to be related to the size of the shock.

Table 5 shows similar responses for groups of providers with a relatively large and small shock.[3] Thus, these results do not support the income effects. Note that we cannot interpret these estimates as causal. First, unobserved heterogeneity may be large since we do not observe other possible activities of providers. For example, BUD-providers may differ in their degree of altruism. If altruistic providers received larger shocks than financially motivated providers, then we may also find a positive correlation.

[3]Because there is a large variation in the size of the shocks we also applied several censoring criteria to exclude providers that faced extremely large shocks. However, as this did not alter our estimation results. We also performed the same regression without controls with similar results.

## Appendix F: Robustness analyses

In this section, we perform four additional analyses with treatment duration as the dependent variable and at the treatment episode level and test whether our results are not driven by other mechanisms.

Table 6: Robustness analyses

| Dependent variable: Treatment duration | | | | | |
|---|---|---|---|---|---|
| | All | Direct treatment duration | Antici-pation effects | Recoding effect | Number of days |
| *FFS-providers* | | | | | |
| Baseline ($\beta_1$) | 2.1 | 2.1 | 2.2 | 1.8 | 1.9 |
| | (0.4) | (0.4) | (0.4) | (0.6) | (0.3) |
| | [1.4, 2.8] | [1.4, 2.8] | [1.4,3.1] | [0.7, 2.9] | [1.3,2.4] |
| Response 2012-2013 ($\beta_2$) | 2.1 | 3.3 | 0.9 | 1.8 | 1.0 |
| | (0.9) | (0.9) | 1.5 | (1.2) | (0.7) |
| | [0.4, 3.7] | [1.5,5.2] | [-2.0,3.7] | [-0.6,4.3] | [-0.5,2.5] |
| Controls | yes | yes | yes | yes | yes |
| Number of observations | 252,776 | 252,776 | 210,978 | 63,440 | 252,776 |
| $R^2$ | 0.247 | 0.224 | 0.246 | 0.246 | 0.199 |
| *BUD-providers* | | | | | |
| Baseline ($\beta_1$) | 1.2 | 0.8 | 0.9 | 2.4 | -0.9 |
| | (0.4) | (0.4) | (0.5) | (0.5) | (0.3) |
| | [0.6, 2.0] | [0.0, 1.6] | [-0.1, 1.9] | [1.3,3.6] | [-1.5, -0.3] |
| Response 2012-2013 ($\beta_2$) | 7.9 | 8.1 | 10.3 | 5.5 | 3.4 |
| | (1.0) | (1.1) | (2.2) | (2.1) | (0.7) |
| | [5.9, 10.0] | [5.9, 10.2] | [5.8,14.7] | [1.3,9.7] | [2.1, 4.8] |
| Controls | yes | yes | yes | yes | yes |
| Number of observations | 3,892,093 | 3,892,093 | 3,277,931 | 979,594 | 3,892,093 |
| $R^2$ | 0.227 | 0.186 | 0.228 | 0,259 | 0.121 |

Notes: In this table we present the estimates of the two $\beta$'s (see Section **??**), as a percentage differences from baseline. Below the $\beta$'s we report the standard errors and 95% confidence intervals. All estimations included all case-mix controls

First, we test whether the change in treatment duration was different for direct or indirect time with the patient. Total treatment duration is the sum of direct, i.e. face-to-face time with patients, and indirect time, i.e. administrative and other tasks. Therefore, we perform our estimations of model (**??**) for direct treatment time only. In Table 6 we report the results in the column "Direct treatment duration". The results are very similar to the main results, as presented in the column "All', which suggests that both types of providers increased their direct and indirect time in the same proportion.

Second, we test whether anticipation effects are important. The policy reform was legally announced by the government in June 2011 and providers may have anticipated before 2012. For example, they may have started new treatments already at the end of 2011, for example to prevent patients from paying a deductible in 2012. Moreover, it may have taken some time before providers became accustomed to the new situation in 2012. Therefore, we leave out all observations from July 2011 until June 2012 in our regression. The average treatment duration responses become somewhat smaller for FFS-providers and larger for BUD-providers.

Third, we perform a second test to see whether recoding influences our results. As we discussed in the main text the diagnoses "adjustment" and "V-codes" were excluded from the basic benefit package. Table 3 and 2 show that for both types of providers the number of treatment episodes drop drastically in 2012-2013. Hence, some providers may have recoded these patients to other diagnoses, such as "mood", in order to prevent that patients needs to pay the full fee for the treatment. If the average treatment duration of the new diagnoses is smaller/larger then for "adjustment" and "V-codes" then this will bias the effect of $\beta_2$. One way to study this effect is to look at diagnoses of severe patients that are not likely to be recoded. In the case of BUD-providers, for example, we find for patients with a severe early childhood disorders (about annually 50,000 observations) an average percentage change of treatment duration ($\beta_2$) of 5.9%. For severe delirium, dementia and other cognitive problems (about annually 15,000 observations) we find 8.9%. This suggests that our results are unlikely to be driven strongly by recoding or other changes in the composition of patients. Table 6 shows the regression results for the quartile providers that performed the fewest number of "adjustment" and "V-codes" treatments during 2008 and 2011. Although the size of the demand shock is somewhat smaller for these providers, the size of the shock is due to the deductible of 200 euros still considerable. For example, for BUD-providers we find in 2012, compared to 2011, a drop in the number of treatment episodes of 14% and for FFS-providers of 21%. The results in the column "Recoding effect" in Table 6 show no differences for FFS-providers compared to the results in the first column. For BUD-providers we find a slightly smaller significant average increase of 5.5%. This smaller effect may be partly related to the smaller demand shock or to the relatively large baseline trend of 2.4%.

Fourth, we test whether providers treated patient longer when we measure treatment duration in the number of days, instead of minutes. We measure the number of days as the difference between the first day and the last day that a patient visits a provider (as recorded in the DBC). When providers receive fewer patients the remaining patients can, theoretically, be treated in fewer days. However, since we find longer treatment durations after the shock, we might also find an increase in the number of days after the shock. The results are shown in the column "Number of days". The results indicate an increase in the number of days after the shock but the percentages are smaller than compared to treatment duration in minutes.

## References


Douven, R., Remmerswaal, M. and Zoutenbier, R. (2019), 'Do altruistic providers have better treatment outcomes?', *Journal of Human Resources* **54**(2), 310–341.

Dutch Healthcare Authority (2014), Marktscan en beleidsbrief geestelijke gezondheidszorg: Betaalbaarheid en wachttijden 2009-2014, Technical report, Dutch Healthcare Authority.

Dutch Healthcare Authority (2016), Marktscan ggz 2016, Technical report, Dutch Healthcare Authority.

Yip, W. (1998), 'Physician responses to medical fee reductions: Changes in the volume and intensity of supply of coronary artery bypass graft (cabg) surgeries in the medicare and private sectors', *Journal of Health Economics* **17**(6), 675–700.
